# A Ca^2+^ channel differentially regulates Clathrin-mediated and activity-dependent bulk endocytosis

**DOI:** 10.1371/journal.pbio.2000931

**Published:** 2017-04-17

**Authors:** Chi-Kuang Yao, Yu-Tzu Liu, I-Chi Lee, You-Tung Wang, Ping-Yen Wu

**Affiliations:** 1 Institute of Biological Chemistry, Academia Sinica, Nankang, Taipei, Taiwan; 2 Neuroscience Program in Academia Sinica, Academia Sinica, Nankang, Taipei, Taiwan; 3 Institute of Biochemical Sciences, College of Life Science, National Taiwan University, Taipei, Taiwan; UT Southwestern Medical Center, United States of America

## Abstract

Clathrin-mediated endocytosis (CME) and activity-dependent bulk endocytosis (ADBE) are two predominant forms of synaptic vesicle (SV) endocytosis, elicited by moderate and strong stimuli, respectively. They are tightly coupled with exocytosis for sustained neurotransmission. However, the underlying mechanisms are ill defined. We previously reported that the Flower (Fwe) Ca^2+^ channel present in SVs is incorporated into the periactive zone upon SV fusion, where it triggers CME, thus coupling exocytosis to CME. Here, we show that Fwe also promotes ADBE. Intriguingly, the effects of Fwe on CME and ADBE depend on the strength of the stimulus. Upon mild stimulation, Fwe controls CME independently of Ca^2+^ channeling. However, upon strong stimulation, Fwe triggers a Ca^2+^ influx that initiates ADBE. Moreover, knockout of rodent *fwe* in cultured rat hippocampal neurons impairs but does not completely abolish CME, similar to the loss of *Drosophila fwe* at the neuromuscular junction, suggesting that Fwe plays a regulatory role in regulating CME across species. In addition, the function of Fwe in ADBE is conserved at mammalian central synapses. Hence, Fwe exerts different effects in response to different stimulus strengths to control two major modes of endocytosis.

## Author summary

The arrival of an action potential at the nerve end induces synaptic vesicle (SV) exocytosis to allow the release of chemical neurotransmitters and the rapid transmission of signals. SV endocytosis is in turn elicited in order to rapidly replenish the vesicle pool in neurons. Therefore, tight coupling between exocytosis and endocytosis within these cells maintains constant synaptic transmission. Exocytosis and intracellular Ca^2+^ elevation are known to be key prerequisites for the two main modes of SV endocytosis, Clathrin-mediated endocytosis (CME) and activity-dependent bulk endocytosis (ADBE), which are primarily triggered by moderate and strong nerve stimuli, respectively. However, how these two events cooperate to trigger endocytosis upon exocytosis remains unclear. In this study, we show that Flower (Fwe), an SV-associated Ca^2+^ channel, plays a significant role in these processes in *Drosophila*. Upon mild stimulation, we find that Fwe is transferred from the SV to endocytic zones to promote CME independently of Ca^2+^ channeling. In contrast, upon intense stimulation Fwe triggers Ca^2+^ influxes that elicit ADBE. In addition, we find that Fwe promotes CME and ADBE also in mammalian central synapses, revealing a conserved role for Fwe in endocytosis. We conclude that the Fwe channel exerts two different functions in response to two different stimuli to govern distinct modes of synaptic vesicle retrieval, thereby coupling exocytosis to endocytosis.

## Introduction

In the presynaptic terminal, continuous release of synaptic vesicles (SVs) results in vesicle pool depletion, plasma membrane expansion, and SV protein overloading at the release site [1]. Endocytosis is therefore tightly coupled to exocytosis [2]. Among the different modes of SV endocytosis, Clathrin-mediated endocytosis (CME) and activity-dependent bulk endocytosis (ADBE) are well characterized [3,4]. In mild stimulation paradigms, CME is the prevalent mode of retrieving exocytic SVs in the form of a single SV [5,6]. In intense stimulation paradigms, however, ADBE promotes the uptake of large pieces of fused membranes in bulk endosomes or cisternae [7,8]. Small SVs are then formed from these membranous structures.

SV exocytosis is a prerequisite for CME and ADBE initiation [2], indicating that specific SV cargoes, an exocytic protein complex, or both are needed to trigger both modes of endocytosis. Indeed, Synaptotagmin, the SV Ca^2+^ sensor for exocytosis [9], and components of the soluble NSF attachment protein receptor (SNARE) complex play crucial roles in CME [10–14]. Moreover, recent studies have identified vesicle-associated membrane protein 4 (VAMP4), a vesicle-associated SNARE (v-SNARE) protein, as a selective SV cargo for ADBE. Interestingly, VAMP4 is responsible for the formation of ADBE as well [15]. Thus, SV proteins encode components that retrieve SV membrane in newly formed vesicles as well as coordinate the nature of the formation of SV endocytosis.

An increased local Ca^2+^ concentration in the presynaptic terminal is necessary not only for exocytosis but also for endocytosis [16–20]. Moreover, the Calmodulin/Calcineurin complex was proposed to function as a Ca^2+^ sensor for CME and ADBE [19,21,22]. Therefore, Ca^2+^/Calcineurin likely acts as a universal signal that elicits most forms of the SV retrieval [2]. At the rat Calyx of Held, in addition to a role in triggering exocytosis, a high, transient Ca^2+^ influx via a voltage-gated Ca^2+^ channel (VGCC) triggers CME [18,23]. However, the Ca^2+^ channel for triggering ADBE is unknown.

Our previous genetic screen in flies identified Flower (Fwe), an evolutionarily conserved transmembrane protein [24]. Fwe forms a Ca^2+^-permeable channel when expressed in heterologous cells or when incorporated into proteoliposomes. This protein localizes to SVs, and, upon SV release, Fwe is transferred to the periactive zone, where it triggers CME, thereby coupling exocytosis to CME. In the present study, we show that Fwe initiates ADBE as well. Intriguingly, the effects of Fwe on CME and ADBE depend on the strength of the stimulus. We found that the function of Fwe for regulating CME does not involve Ca^2+^ channeling. Instead, upon intense stimulation, Fwe triggers a Ca^2+^ influx that elicits ADBE. Lastly, when we removed *ratFwe* in cultured rat hippocampal neurons through clustered regularly interspaced short palindromic repeats (CRISPR)/ CRISPR-associated protein 9 (Cas9) technology, CME is impaired but not completely blocked, similar to the defect caused by the *Drosophila fwe* mutation. Furthermore, our data reveal that RatFwe is also involved in the induction of ADBE at mammalian central synapses. In summary, the Fwe channel exerts two different functions in response to two different stimuli that govern distinct modes of SV retrieval, thereby coupling exocytosis to endocytosis.

## Results

### Fwe promotes CME independently of Ca^2+^channeling

We previously reported that the Fwe Ca^2+^ channel promotes CME in the synaptic boutons of *Drosophila* neuromuscular junctions (NMJs) [24]. To investigate whether the Ca^2+^ influx via Fwe plays a direct role in CME, we utilized the FweE79Q mutant whose channel activity is severely impaired [24] and assessed the ability to rescue CME defects associated with *fwe* mutants. We expressed *UAS*-*flag-fwe-HA* and *UAS-flag-fweE79Q-HA* with *nSyb-GAL4*, a pan-neuron *GAL4* driver, in a strong loss of *fwe* background (*fwe*^*DB25*^*/fwe*^*DB56*^) (S1A and S1B Fig) [24]. α-HA antibody staining was used to examine the distribution and expression of Fwe proteins in boutons. α-horse radish peroxidase (HRP) antibody staining labels the insect neuronal membranes, thereby outlining presynaptic compartments [25]. Both the wild-type Fwe and FweE79Q proteins are evenly distributed in boutons (S1A and S1B Fig). We then introduced a genomic *HA*-tagged *fwe* transgene to estimate the relative expression levels of *UAS* transgenes versus endogenous Fwe protein (S1C–S1F Fig). The proteins are expressed at ~50% of the endogenous Fwe protein level in type Ib NMJ boutons (S1L–S1N Fig), whereas their expressions in type Is NMJ boutons correspond to ~80% of the endogenous Fwe protein.

**Fig 1:**
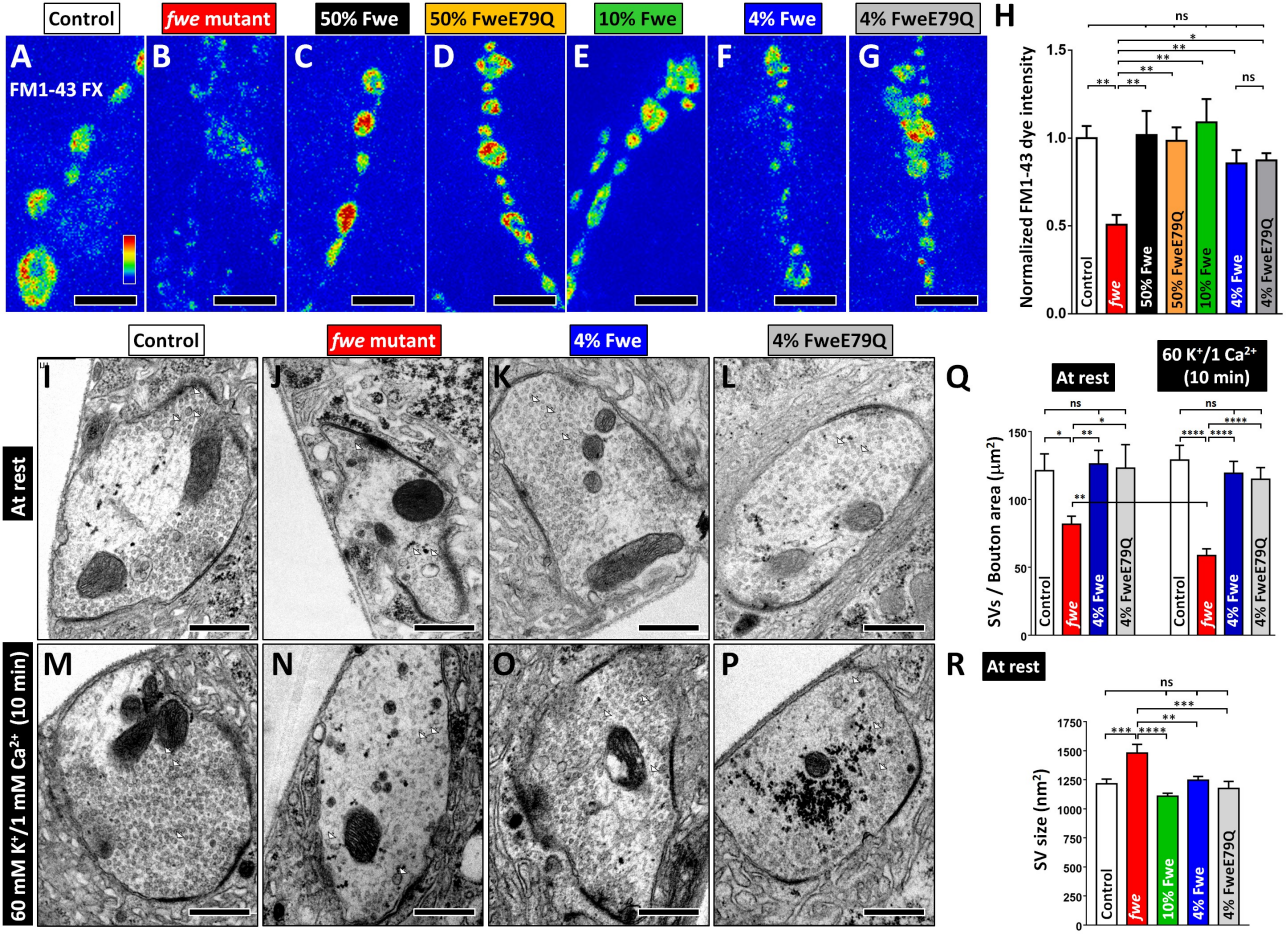
Fwe controls Clathrin-Mediated Endocytosis (CME) in a channel-independent manner. (A–G) Confocal Z-projection images of neuromuscular junction (NMJ) boutons labeled with fixable FM1-43 dye were obtained from *FRT80B* control larvae (A), *fwe* mutant larvae (*fwe*^*DB25*^*/fwe*^*DB56*^, B), 50% Fwe-rescued larvae (*nSyb* > *flag-fwe-HA* in *fwe*^*DB25*^*/fwe*^*DB56*^, C), 50% FweE79Q-rescued larvae (*nSyb* > *flag-fweE79Q-HA* in *fwe*^*DB25*^*/fwe*^*DB56*^, D), 10% Fwe-rescued larvae (*elav* > *flag-fwe-HA* in *fwe*^*DB25*^*/fwe*^*DB56*^, E), 4% Fwe-rescued larvae (*nSyb(w)* > *flag-fwe-HA* in *fwe*^*DB25*^*/fwe*^*DB56*^, F) and 4% FweE79Q-rescued larvae (*nSyb(w)* > *flag-fweE79Q-HA* in *fwe*^*DB25*^*/fwe*^*DB56*^, G). FM1-43 dye uptake was elicited by 1-min 90 mM K^+^/0.5 mM Ca^2+^ stimulation. (H) The absolute dye fluorescence intensities in the boutons were measured and normalized to the average value of controls. Loss of *fwe* significantly impairs dye uptake, which can be restored by expressing different levels of wild-type Fwe or FweE79Q protein. Type Ib boutons derived from A2 muscles 6/7 were counted, and NMJs (control, *n* = 8; *fwe* mutant, *n* = 6; 50% Fwe, *n* = 6; 50% FweE79Q, *n* = 6; 10% Fwe, *n* = 6; 4% Fwe, *n* = 8; and 4% FweE79Q, *n* = 6) derived from 5 larvae for each genotype were analyzed. (I–P) Transmission electron microscopy (TEM) images of NMJ boutons were obtained from larvae of the indicated genotypes. They were processed under the resting condition (10-min incubation in 5 mM K^+^/0 mM Ca^2+^ solution, I–L) or after 10-min 60 mM K^+^/1 mM Ca^2+^ stimulation (M–P). White arrows indicate individual synaptic vesicles (SVs). (Q) Data quantifications of SV density. A low SV number was found in *fwe* mutants, which is corrected in the presence of 4% Fwe and 4% FweE79Q. Ten-minute 60 mM K^+^/1 mM Ca^2+^ stimulation further lowers the SV density in *fwe* mutants. Type Ib boutons (at rest: control, *n* = 14; *fwe* mutant, *n* = 23; 4% Fwe, *n* = 19; and 4% FweE79Q, *n* = 13. Ten-minute 60 mM K^+^/1 mM Ca^2+^ stimulation: control, *n* = 13; *fwe* mutant, *n* = 36; 4% Fwe, *n* = 14; and 4% FweE79Q, *n* = 24) derived from 3 larvae for each genotype were analyzed. (R) Data quantifications of SV size. Enlarged SV size was found in *fwe* mutants, which is corrected in the presence of 10% Fwe, 4% Fwe, or 4% FweE79Q. SVs (control, *n* = 1,675; *fwe* mutants, *n* = 780; 10% Fwe, *n* = 2,196; 4% Fwe, *n* = 1,243; and 4% FweE79Q, *n* = 857) obtained from ≥10 type Ib boutons were analyzed. Boutons were derived from ≥3 larvae for each genotype. All statistical analyses used a one-way ANOVA test, except the comparison of the SV number at rest and after stimulation, which was performed using a Student’s *t* test. *p*-Value: ns, not significant; *, *p* < 0.05; **, *p* < 0.01; ***, *p* < 0.01; ****, *p* < 0.001. Error bars indicate the standard error of the mean. Scale bar: 5 μm in A–G; 500 nm in I–P. The underlying data can be found in S1 Data.

To estimate the efficacy of CME, we performed the FM1-43 dye uptake assay with moderate stimuli, i.e., 1-min 90 mM K^+^/0.5 mM Ca^2+^ and 10-min 60 mM K^+^/1 mM Ca^2+^ stimulations. The experimental paradigm is shown in S2A Fig. Both stimulation paradigms significantly elicit dye uptake in wild-type control larvae when compared to a resting paradigm (10-min incubation in 5 mM K^+^/0 mM Ca^2+^ solution) (S2B–S2E Fig). We then performed a transmission electron microscopy (TEM) assay to assess the formation of bulk cisternae, a hallmark of ADBE [2,8]. No bulk cisternae were induced under these conditions (S2F–S2I Fig), showing that the strength of these stimuli is mild, which predominantly promotes CME. Upon 1-min 90 mM K^+^/0.5 mM Ca^2+^ stimulation, loss of *fwe* impairs FM1-43 dye uptake (Fig 1A, 1B and 1H). It is possible that either a defect in SV endocytosis or exocytosis would reduce FM1-43 dye uptake in this case. To test the role of Fwe in SV exocytosis, we performed the FM1-43 dye loading/unloading assay. The experimental paradigm is indicated in S3A Fig. Both the control and *fwe* mutant boutons were subjected to 5-min 90 mM K^+^/0.5 mM Ca^2+^ stimulation, which labels the SV pool with FM1-43 dye (S3B and S3D Fig). Subsequently, 1-min stimulation with the same solution releases the SVs and unloads the dye from SVs (S3C and S3E Fig). The strength of SV exocytosis is correlated with the FM1-43 dye unloading efficiency ([(F_load_—F_unload_) / F_load_]). While the dye loading is significantly reduced when *fwe* is lost (S3B, S3D and S3F Fig), the dye unloading efficiencies of controls and *fwe* mutants are comparable (S3G Fig), indicating that Fwe plays a marginal or no role in SV exocytosis. Hence, the FM1-43 dye uptake deficit associated with *fwe* mutants mainly results from a defect in CME.

Reduced FM1-43 dye uptake in *fwe* mutants is completely rescued by the reintroduction of 50% Fwe protein (Fig 1C and 1H). However, a similar rescue was also observed when 50% FweE79Q is present (Fig 1D and 1H). Although the channel function of FweE79Q is mostly absent when analyzed in fly salivary glands [24], the remaining channel activity in this mutant might be sufficient to promote CME if enough proteins are present in the boutons. We therefore determined the minimal Fwe level required for CME to verify the channel function of Fwe. After surveying numerous *GAL4* lines, *elav-GAL4* and *nSyb(w)-GAL4* were found to drive the expression of the transgenes at approximately 0% and 4% of the endogenous Fwe level, respectively (S1G–S1J, S1M and S1N Fig). As shown in Fig 1E, 1F and 1H, boutons expressing 10% or 4% Fwe can take up FM1-43 dye efficiently. We noted that the dye uptake with 4% Fwe expression is marginally reduced when compared to other Fwe-expressing larvae, indicating that this level of Fwe expression is near the minimal level for efficient CME.

Next, we assessed CME in 4% FweE79Q-expressing boutons (S1J, S1K, S1M and S1N Fig). Intriguingly, the efficiency of the dye uptake is not different between 4% Fwe and 4% Fwe E79Q-expressing boutons (Fig 1F–1H), suggesting that CME can occur despite the lack of a significant Ca^2+^ influx via Fwe. We previously showed that loss of *fwe* results in a reduced SV number and enlarged SVs [24]. To examine the changes in SV ultrastructure, we performed TEM. The wild-type control bouton under the resting condition contains numerous SVs (Fig 1I), whereas the number of SVs is decreased upon loss of *fwe* (Fig 1J and 1Q). This low SV number worsens following 10-min 60 mM K^+^/1 mM Ca^2+^ stimulation (Fig 1M, 1N and 1Q). Either 4% Fwe or 4% FweE79Q expression rescues this SV loss (Fig 1K, 1L and 1O–1Q). In addition, enlarged SV sizes associated with *fwe* mutants are normalized under both expression conditions (Fig 1R). Hence, these data indicate that Fwe triggers CME independent of Ca^2+^ channeling.

### Fwe initiates ADBE upon intense stimulation

**Fig 2:**
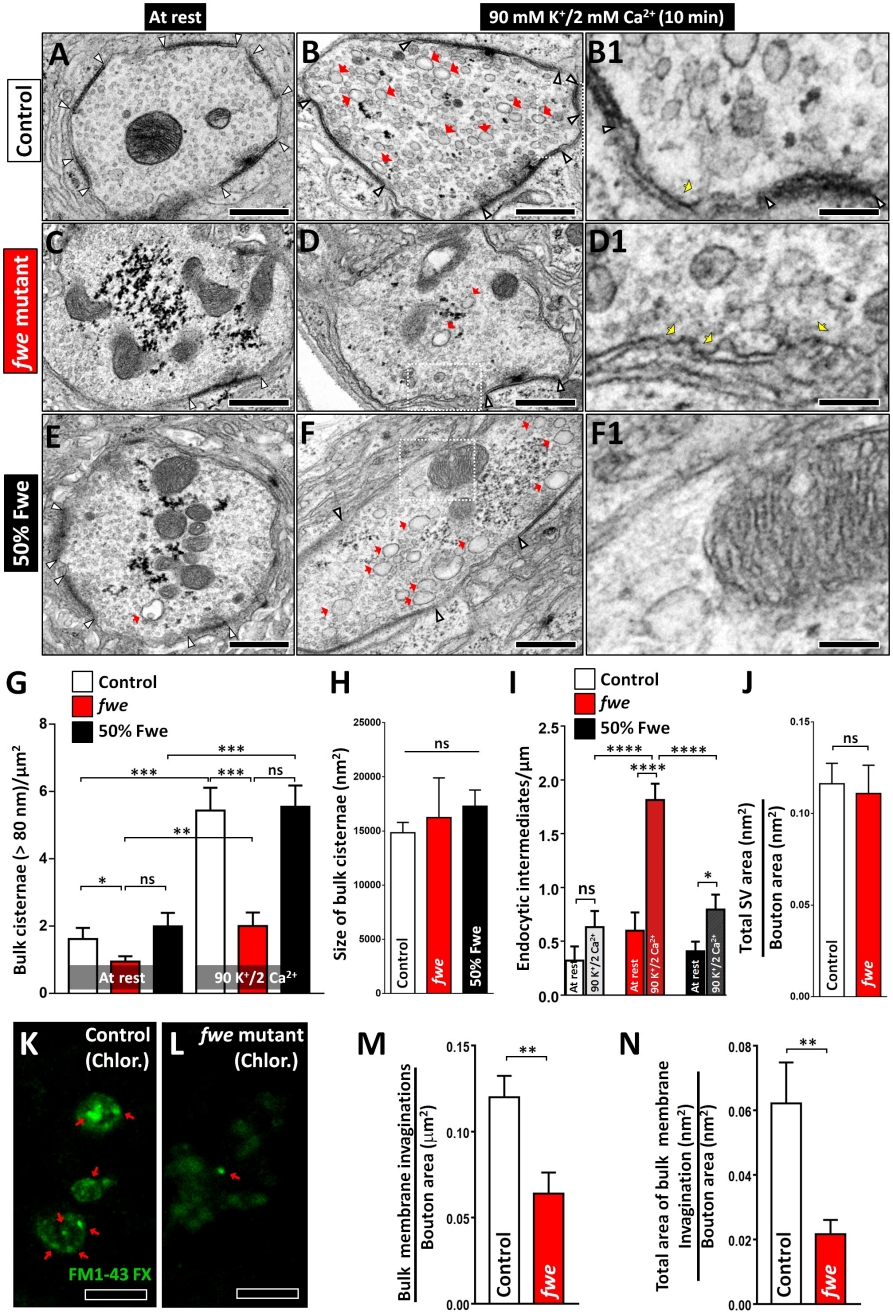
Loss of *fwe* impairs Activity-Dependent Bulk Endocytosis (ADBE) in high K^+^ stimulation. (A–F) Transmission electron microscopy (TEM) images of neuromuscular junction (NMJ) boutons were obtained from *FRT80B* control larvae (A, B, and B1), *fwe* mutant larvae (*fwe*^*DB25*^*/fwe*^*DB56*^; C, D, and D1), and 50% Fwe-rescued larvae (E, F, and F1). Samples were processed under the resting condition (10-min incubation in 5 mM K^+^/0 mM Ca^2+^ solution; A, C, and E) or after 10-min 90 mM K^+^/2 mM Ca^2+^ stimulation (B–B1, D–D1, and F–F1). Red arrows indicate bulk cisternae larger than 80 nm in diameter. White arrowheads indicate the border of the active zone. Loss of *fwe* impairs the formation of high K^+^-induced bulk cisternae. This deficit is rescued by 50% Fwe expression. Enlarged images of B, D, and F (white dashed boxes) are shown in B1, D1, and F1. Yellow arrows indicate endocytic intermediates formed around the periactive zone. (G) Data quantifications of the number of bulk cisternae per bouton area. The data in resting controls are derived from S2I Fig. Type Ib boutons (at rest: control, *n* = 17; *fwe* mutant, *n* = 31; and 50% Fwe, *n* = 11. Ten-minute 90 mM K^+^/2 mM Ca^2+^ stimulation: control, *n* = 29; *fwe* mutant, *n* = 22; and 50% Fwe, *n* = 27) derived from ≥3 larvae for each genotype were analyzed. (H) Data quantifications of the size of bulk cisternae. Bulk cisternae (control, *n* = 275; *fwe* mutant, *n* = 38; and 50% Fwe, *n* = 153) obtained from ≥20 type Ib boutons were analyzed. Boutons were derived from ≥3 larvae for each genotype. (I) Data quantifications of the number of endocytic intermediates per periactive zone length. Following high K^+^ stimulation, more endocytic intermediates were observed in *fwe* mutant boutons when compared to control and 50% Fwe-rescued boutons. Type Ib boutons (at rest: control, *n* = 13; *fwe* mutant, *n* = 25; and 50% Fwe, *n* = 20. Ten-minute 90 mM K^+^/2 mM Ca^2+^ stimulation: control, *n* = 26; *fwe* mutant, *n* = 18; and 50% Fwe, *n* = 25) derived from ≥3 larvae for each genotype were analyzed. (J) Data quantifications of the ratio of total synaptic vesicle (SV) area to bouton area. Type Ib boutons (control, *n* = 14; and *fwe* mutant, *n* = 16) derived from 3 larvae for each genotype were analyzed. (K–L) Confocal Z-projection images of NMJ boutons labeled with fixable FM1-43 dye were obtained from *FRT80B* controls (K) and *fwe* mutants (L). Larval fillets were treated with 200 μM chlorpromazine for 30 min, followed by 10-min 90 mM K^+^/2 mM Ca^2+^/200 μM chlorpromazine stimulation in the presence of FM1-43 dye. Bulk membranous invaginations loaded with high levels of FM1-43 dye are indicated by red arrows. (M–N) Data quantifications of the number of bulk cisternae per bouton area and the ratio of total area of bulk membrane invaginations to bouton area. After chlorpromazine treatment, bulk membrane invaginations are impeded upon loss of *fwe*. Type Ib boutons derived from A2/A3 muscles 4 or 6/7 were counted, and NMJs (control, *n* = 25; *fwe* mutant, *n* = 23) derived from 5 larvae for each genotype were analyzed. A Student’s *t* test was used for analyses in G, I, J, M, and N. A one-way ANOVA test was used in H. *p*-Value: ns, not significant; *, *p* < 0.05; **, *p* < 0.01; ***, *p* < 0.01; ****, *p* < 0.001. Error bars indicate the standard error of the mean. Scale bar: 500 nm in A–F; 125 nm in B1, D1, and F1; 5 μm in K–L. The underlying data can be found in S1 Data.

Upon mild stimulation, CME retrieves the membrane that corresponds in size to an SV. In contrast, in response to intense stimulation, ADBE takes up large quantities of fused SVs from the plasma membrane to form bulk cisternae. It has been shown that both SV exocytosis and intracellular Ca^2+^ elevation are essential for ADBE to proceed around the periactive zones [7,18,26]. This raises the possibility that Fwe may play a role in ADBE. High K^+^ and Ca^2+^-containing solutions have been widely used to elicit ADBE at several different synapses, including fly NMJ boutons [8,27–30]. To assess the role of Fwe in ADBE, we applied 10-min 90 mM K^+^/2 mM Ca^2+^ stimulation to induce ADBE and examine the formation of bulk cisternae using a TEM assay. The TEM image of control boutons reveals numerous cisternae (>80 nm in diameter, red arrows) elicited by this stimulation paradigm (Fig 2A, 2B and 2G). These processes, however, are dramatically suppressed by loss of *fwe* (Fig 2C, 2D and 2G). In unstimulated conditions, bulk cisternae are also less abundant in *fwe* mutants than in controls (Fig 2G). This ADBE defect indeed results from the *fwe* mutation, as 50% Fwe expression rescues this ADBE phenotype (Fig 2E–2G). Interestingly, the average size of the few bulk cisternae observed in *fwe* mutants is comparable to that observed in control boutons (Fig 2H), suggesting that Fwe acts at the initiation step of ADBE rather than during a late membrane invagination process. Furthermore, following high K^+^ stimulation, the accumulation of early endocytic intermediates was observed around the periactive zone in *fwe* mutant boutons (Fig 2D–2D1 and 2I, yellow arrows) when compared to wild-type controls and 50% Fwe-rescued larvae (Fig 2B–2B1, 2F–2F1 and 2I). Since optimal SV exocytosis is shown as a prerequisite for triggering ADBE [7,26], we therefore estimated the total SV area per bouton area under the resting condition. No difference between controls and *fwe* mutants was found (Fig 2J), showing that the ADBE defect associated with *fwe* mutants is not due to insufficient supply of exocytic SV membrane upon stimulation. Moreover, following 90 mM K^+^/2 mM Ca^2+^ stimulation, the strength of SV exocytosis determined by the FM1-43 dye loading/unloading assay is comparable between controls and *fwe* mutants (S3H–S3N Fig). Collectively, these results reveal that Fwe is responsible for initiating ADBE during intense activity stimulation.

Acute inactivation of the components involved in CME, such as Clathrin, AP180, and Dynamin, elicits bulk membrane invaginations [31–34], suggesting that CME suppresses ADBE or that ADBE is the result of membrane expansions. To assess the role of Fwe in this process, we treated larvae with 200 μM chlorpromazine to inhibit Clathrin coat assembly [31], followed by 10-min 90 mM K^+^/ 2 mM Ca^2+^ stimulation in the presence of FM1-43 dye. As shown in Fig 2K–2N, large membranous invaginations enriched with FM1-43 dye were detected in the controls. In contrast, these structures decrease upon loss of *fwe*. In summary, these results reinforce the functional importance of Fwe in ADBE.

### The Ca^2+^ influx via Fwe sustains presynaptic Ca^2+^ levels during strong stimulation

**Fig 3:**
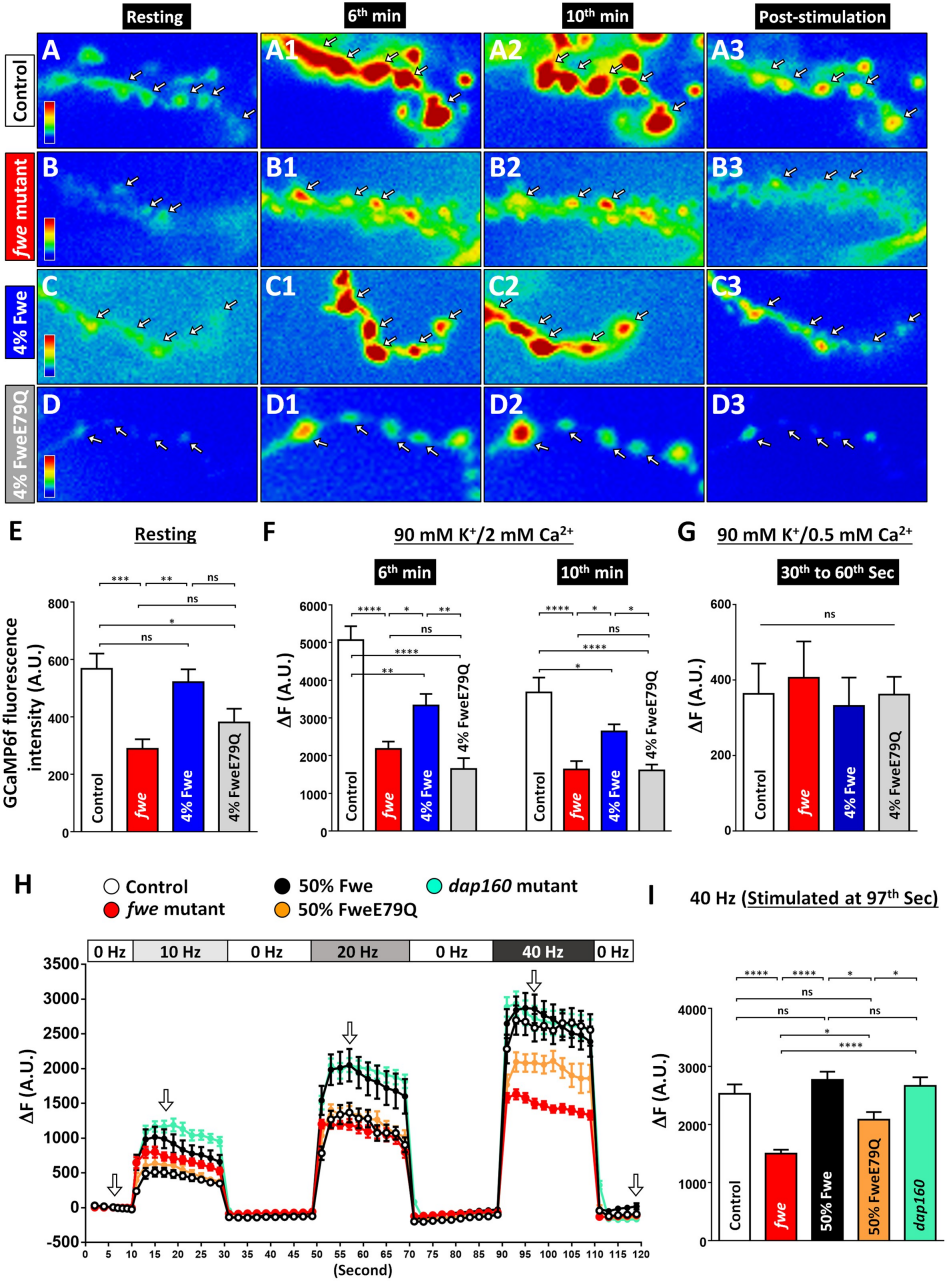
Presynaptic Ca^2+^ levels are perturbed in *fwe* mutants upon strong stimulation. (A–E) Pseudocolored GCaMP6f images of neuromuscular junction (NMJ) boutons were obtained from control larvae (*vglut-lexA/lexAop2-GCaMP6f*, *nSyb(w)-GAL4/+* in *fwe*^*DB25*^*/+*, A), *fwe* mutant larvae (*vglut-lexA/lexAop2-GCaMP6f*, *nSyb(w)-GAL4/+* in *fwe*^*DB25*^*/fwe*^*DB56*^, B), 4% Fwe-rescued larvae (*vglut-lexA/lexAop2-GCaMP6f*, *nSyb(w)-GAL4/UAS-flag-fwe-RB-HA* in *fwe*^*DB25*^*/fwe*^*DB56*^, C), and 4% FweE79Q-rescued larvae (*vglut-lexA/lexAop2-GCaMP6f*, *nSyb(w)-GAL4/UAS-flag-fweE79Q-RB-HA* in *fwe*^*DB25*^*/fwe*^*DB56*^, D). The *vglut-lexA/lexAop2-GCaMP6f* larvae expressed detectable amounts of GCaMP6f in boutons when the weak *nSyb(w)-GAL4* driver was used to drive expression of *UAS-fwe* transgenes. Therefore, the use of this binary system allowed us to compare the Ca^2+^ imaging results between controls, *fwe* mutants, 4% Fwe-rescued larvae, and 4% FweE79Q-rescued larvae. Boutons were subjected to 10-min 90 mM K^+^/2 mM Ca^2+^ stimulation. Representative GCaMP6f images were captured at rest, in the 6th min, in the 10th min, and after stimulation. White arrows indicate type Ib boutons. (E) Data quantifications of the absolute unit (A.U.) of the resting GCaMP6f fluorescence intensity. Loss of *fwe* impairs the resting Ca^2+^ levels, which is normalized by expressing 4% Fwe but not 4% FweE79Q. Type Ib boutons of A3 muscles 6/7 were counted, and NMJs (control, *n* = 13; *fwe* mutant, *n* = 16; 4% Fwe, *n* = 17; and 4% FweE79Q, *n* = 14) derived from ≥6 larvae for each genotype were analyzed. (F) Data quantifications of the increases in GCaMP6f fluorescence in the 6th or 10th min. The increase in the intracellular Ca^2+^ level in response to high K^+^ stimulation is perturbed by loss of *fwe*. The expression of 4% Fwe but not 4%FweE79Q partially rescues these defects. Type Ib boutons of A3 muscles 6/7 were counted, and NMJs (6th min: control, *n* = 5; *fwe* mutant, *n* = 6; 4% Fwe, *n* = 8; 4% FweE79Q, *n* = 6. 10th min: control, *n* = 7; *fwe* mutant, *n* = 6; 4% Fwe, *n* = 10; and 4% FweE79Q, *n* = 6) derived from ≥5 larvae for each genotype were analyzed. (G) Larvae of the indicated genotypes were subjected to 1-min 90 mM K^+^/0.5 mM Ca^2+^ stimulation. The GCaMP6f images were taken between 30–60 s. The increase in GCaMP6f fluorescence is shown. The Ca^2+^ increase is similar among all genotypes. Type Ib boutons of A3 muscles 6/7 were counted, and NMJs (control, *n* = 6; *fwe* mutant, *n* = 6; 4% Fwe, *n* = 6; and 4% FweE79Q, *n* = 6) derived from 6 larvae for each genotype were analyzed. (H) The time-course traces of the changes in GCaMP6f fluorescence in NMJ boutons in response to electric stimuli. The images were captured from control larvae (*nSyb*-*GAL4/UAS*-*GCaMP6f* in *fwe*^*DB25*^*/+*, white circle), *fwe* mutant larvae (*nSyb*-*GAL4/UAS*-*GCaMP6f* in *fwe*^*DB25*^*/fwe*^*DB56*^, red circle), 50% Fwe-rescued larvae (*nSyb*-*GAL4/UAS*-*GCaMP6f*/*UAS*-*flag-fwe-RB-HA* in *fwe*^*DB25*^*/fwe*^*DB56*^, black circle), 50% FweE79Q-rescued larvae (*nSyb*-*GAL4/UAS*-*GCaMP6f*/*UAS*-*flag-fweE79Q-RB-HA* in *fwe*^*DB25*^*/fwe*^*DB56*^, orange circle), and *dap160* mutants (*nSyb*-*GAL4/UAS*-*GCaMP6f* in *dap160*^*Δ1*^*/dap160*^*Δ2*^, green circle). Boutons were subjected to train stimulations of 10-, 20-, and 40-Hz-triggered action potentials, with a 20-s rest between train stimuli. White arrows indicate the time points when the representative GCaMP6f images in S5A–S5E Fig were obtained. (I) The average increase in GCaMP6f fluorescence at 97 s is shown. Loss of Fwe or its channel activity does not affect the Ca^2+^ increases evoked at 10 Hz and 20 Hz but impairs the Ca^2+^ increases evoked at 40 Hz. Type Ib boutons of A3 muscles 6/7 were counted, and NMJs (control, *n* = 20; *fwe* mutant, *n* = 16; 50% Fwe, *n* = 14; 50% FweE79Q, *n* = 12; and *dap160* mutant, *n* = 16) derived from ≥6 larvae for each genotype were analyzed. A one-way ANOVA test was used for statistical analysis. *p*-Value: ns, not significant; *, *p* < 0.05; **, *p* < 0.01; ***, *p* < 0.01; ****, *p* < 0.001. Error bars indicate the standard error of the mean. All images were captured in the same scale. The underlying data can be found in S1 Data.

To assess whether Fwe mediates an intracellular Ca^2+^ increase to initiate ADBE upon intense activity stimulation, we expressed the *lexAop2* transgene of the fast-decay version of the genetically encoded Ca^2+^ indicator GCaMP6, GCaMP6f [35], in the presynaptic terminals with *vglut-lexA*, a glutamatergic neuron driver. We have shown previously that *fwe* mutant boutons display low resting Ca^2+^ levels [24]. Similarly, decreased GCaMP6f fluorescence was observed upon loss of *fwe* (Fig 3A, 3B and 3E, white arrows). This indicates a reduction in the resting Ca^2+^ levels, as the expression level of GCaMP6f in boutons is higher in *fwe* mutants than in controls (S4A, S4B and S4E Fig). Next, we stimulated boutons with 90 mM K^+^/2 mM Ca^2+^ solution for 10 min, which elicits ADBE (Fig 2A and 2B), and measured GCaMP6f fluorescence in the 6th and 10th min. In controls, the intracellular Ca^2+^ concentrations in response to stimuli are substantially increased (Fig 3A–3A3 and 3F), whereas loss of *fwe* significantly impedes these Ca^2+^ elevations (Fig 3B–3B3 and 3F). Hence, Fwe sustains presynaptic Ca^2+^ levels upon strong stimulation.

To assess the role of Fwe-derived Ca^2+^ influx in regulating presynaptic Ca^2+^ level, we traced GCaMP6f fluorescence in 4% Fwe- and 4% FweE79Q-expressing boutons under the resting and high K^+^ stimulation conditions. In both conditions, the levels of GCaMP6f are expressed similarly to that in controls (S4C–S4E Fig). We found that 4% Fwe but not 4% FweE79Q expression restores normal resting Ca^2+^ levels in *fwe* mutants (Fig 3C–3E). Furthermore, a defect in high K^+^-induced Ca^2+^ elevation associated with *fwe* mutants is partially reversed by 4% Fwe expression (Fig 3C–3C3 and 3F). In contrast, 4% FweE79Q expression fails to rescue this Ca^2+^ defect (Fig 3D–3D3 and 3F). However, when we examined the presynaptic Ca^2+^ changes following 1-min 90 mM K^+^/0.5 mM Ca^2+^ stimulation, which prevalently elicits CME (S2 Fig), the Ca^2+^ increases among all genotypes are quite similar (Fig 3G). Hence, these results indicate that Fwe triggers a Ca^2+^ influx specifically in response to strong stimuli.

To verify this stimulus-dependent Ca^2+^ channeling of Fwe, we measured the Ca^2+^ increase evoked by electric stimuli in wild-type and *fwe* mutant boutons. To this end, we expressed *UAS-GCaMP6f* with *nSyb-GAL4*. The levels of GCaMP6f in control and *fwe* mutant boutons are comparable (S4F, S4G and S4K Fig). The stimulation paradigms are shown in the top panel of Fig 3H. Upon 10–40 Hz train stimuli, intracellular Ca^2+^ increase in controls correlates with the stimulus strength (S5A–S5A4 Fig and Fig 3H, white circle). In *fwe* mutants (S5B–S5B4 Fig and Fig 3H, red circle), the Ca^2+^ increase at 10 Hz is slightly higher than that in controls, and the Ca^2+^ increase at 20 Hz is similar to that in controls. In contrast, at 40 Hz, loss of *fwe* significantly impairs the evoked Ca^2+^ increase (Fig 3H and 3I). Moreover, 50% Fwe expression normalizes this deficit (S4H, S4K and S5C–S5C4 Figs and Fig 3H and 3I, black circle), whereas a partial restoration by 50% FweE79Q expression was observed (S4I, S4K and S5D–S5D4 Figs and Fig 3H and 3I, orange circle). Furthermore, we observed similar effects on rescuing low resting Ca^2+^ levels associated with *fwe* mutants (S5F Fig). These results support the finding that Fwe does not mediate a Ca^2+^ influx under a moderate stimulation condition that predominantly induces CME. Instead, it conducts a Ca^2+^ influx when neurons undergo intense stimulation.

To rule out that the defect in evoked Ca^2+^ increase may be attributed to slow CME associated with *fwe* mutants, we applied the same stimulation protocol to *dap160* mutants, which exhibit a similar CME defect [36–38]. As shown in S5E–S5E4 Fig and Fig 3H and 3I (green circle), in *dap160* mutant boutons, the Ca^2+^ concentrations at 40 Hz are elevated to wild-type levels, although the Ca^2+^ increase at 10 and 20 Hz is higher than that observed in controls and *fwe* mutants. In addition, the fluorescence level but not the expression level of GCaMP6f under the resting condition is reduced upon loss of *dap160* (S4J, S4K, S5E and S5F Figs), suggesting that *dap160* mutants display low resting Ca^2+^ levels. Therefore, our results argue that a defective CME does not account for presynaptic Ca^2+^ dysregulation in *fwe* mutants. In addition, we found no evidence for the changes in the distribution and expression of Cacophony, the major VGCC located at the active zone (S6 Fig).

### Fwe triggers a Ca^2+^ influx to initiate ADBE during intense activity stimulation

**Fig 4:**
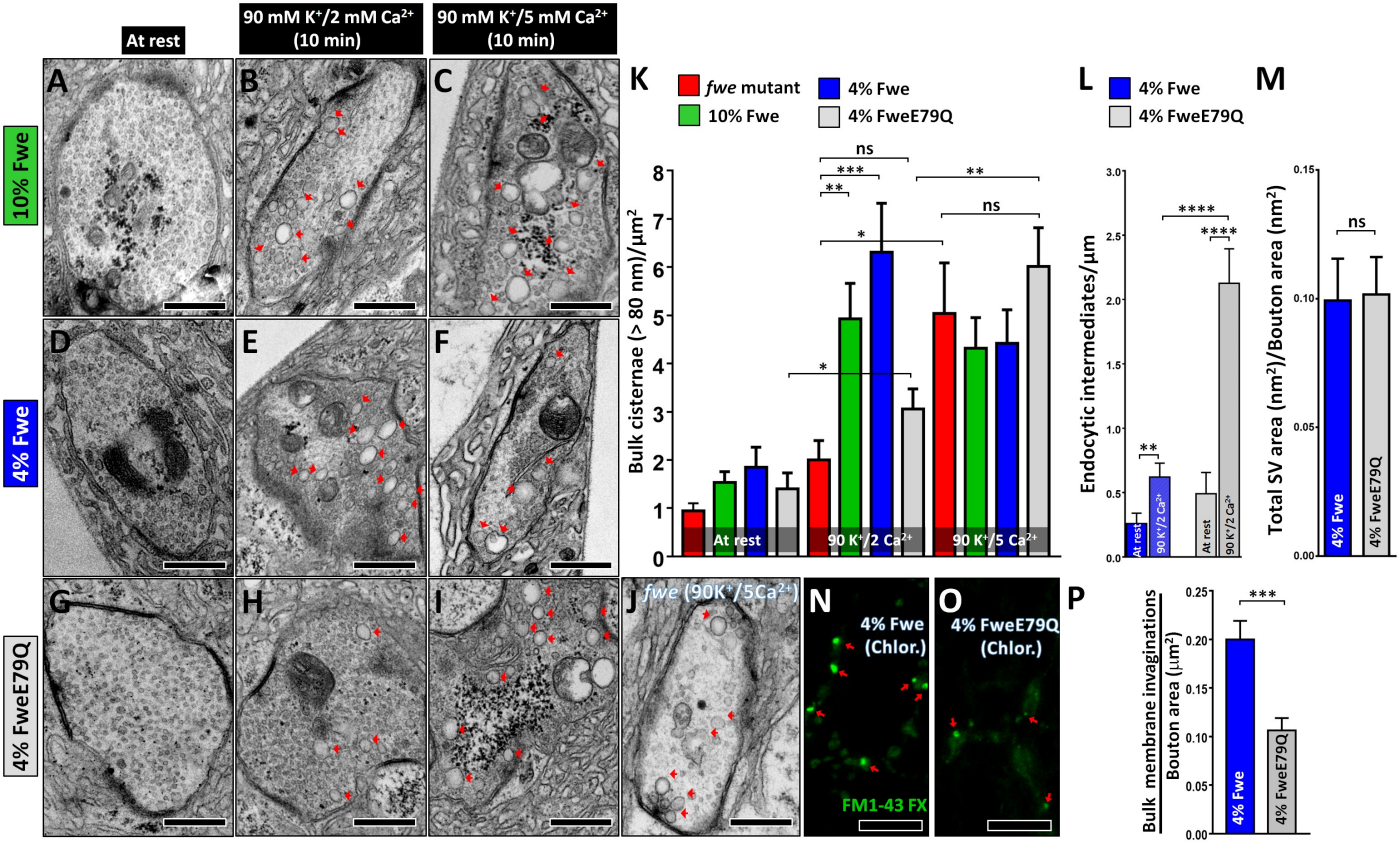
Ca^2+^ influx mediated by Fwe initiates Activity-Dependent Bulk Endocytosis (ADBE). (A–J) Transmission electron microscopy (TEM) images of neuromuscular junction (NMJ) boutons were obtained from larvae of the indicated genotypes. Samples were fixed under the resting condition (10-min incubation in 5 mM K^+^/0 mM Ca^2+^ solution, A, D, and G), after 10-min 90 mM K^+^/2 mM Ca^2+^ stimulation (B, E, and H), or after 10-min 90 mM K^+^/5 mM Ca^2+^ stimulation (C, F, I, and J). Bulk cisternae larger than 80 nm are indicated by red arrows. (K) Data quantifications of the number of bulk cisternae per bouton area. The data for *fwe* mutants at rest and after 90 mM K^+^/2 Ca^2+^ stimulation are derived from Fig 2G. Stimulation with 90 mM K^+^/5 mM Ca^2+^ solution induces a wild-type level of bulk cisternae in 4% FweE79Q-rescued and *fwe* mutant boutons. Type Ib boutons (at rest: *fwe* mutant, *n* = 31; 10% Fwe, *n* = 17; 4% Fwe, *n* = 21; and 4% FweE79Q, *n* = 12. Ten-minute 90 mM K^+^/2 mM Ca^2+^ stimulation: *fwe* mutant, *n* = 22; 10% Fwe, *n* = 27; 4% Fwe, *n* = 23; and 4% FweE79Q, *n* = 26. Ten-minute 90 mM K^+^/5 mM Ca^2+^ stimulation: *fwe* mutant, *n* = 23; 10% Fwe, *n* = 30; 4% Fwe, *n* = 24; and 4% FweE79Q, *n* = 27) derived from ≥3 larvae for each genotype were analyzed. (L) Data quantifications of the number of endocytic intermediates per periactive zone length. Following high K^+^ stimulation, more endocytic intermediates were found in 4% FweE79Q-rescued boutons when compared to 4% Fwe-rescued boutons. Type Ib boutons (at rest: 4% Fwe, *n* = 23; and 4% FweE79Q, *n* = 15. Ten-minute 90 mM K^+^/2 mM Ca^2+^ stimulation: 4% Fwe, *n* = 20; and 4% FweE79Q, *n* = 23) derived from ≥3 larvae for each genotype were analyzed. (M) Data quantifications of the ratio of total synaptic vesicle (SV) area to bouton area. Type Ib boutons (4% Fwe, *n* = 11; and 4% FweE79Q, *n* = 11) derived from 3 larvae for each genotype were analyzed. (N–O) Confocal Z-projection images of NMJ boutons labeled with FM1-43 dye were obtained from 4% Fwe-rescued larvae (N) and 4% FweE79Q-rescued larvae (O). Larval fillets were stimulated with a chlorpromazine-containing solution as indicated in Fig 2K and 2L. Large membrane invaginations enriched with FM1-43 dye are marked by red arrows. (P) Data quantifications of the number of bulk membrane invaginations per bouton area. Chlorpromazine-induced bulk membrane invagination is impaired in 4% FweE79Q-rescued boutons when compared to 4% Fwe-rescued boutons. Type Ib boutons derived from A2/A3 muscles 4 or 6/7 were counted, and NMJs (4% Fwe, *n* = 17; and 4% FweE79Q, *n* = 26) derived from 5 larvae for each genotype were analyzed. A Student’s *t* test was used for statistical analysis. *p*-Value: ns, not significant; *, *p* < 0.05; **, *p* < 0.01; ***, *p* < 0.01; ****, *p* < 0.001. Error bars indicate the standard error of the mean. Scale bar: 500 nm in A–J; 5 μm in N–O. The underlying data can be found in S1 Data.

The above-mentioned results prompted investigations into the role of Fwe-driven Ca^2+^ influx in ADBE. We showed previously that 4% Fwe is sufficient for CME. We therefore addressed if this level of Fwe is sufficient to promote ADBE. When boutons are expressed with 10% or 4% Fwe, ADBE elicited by 10-min 90 mM K^+^/2 mM Ca^2+^ stimulation efficiently produces bulk cisternae (Fig 4A, 4B, 4D, 4E and 4K). Thus, a partial Ca^2+^ influx by 4% Fwe (Fig 3F) is sufficient for initiating ADBE. Consistently, 50% FweE79Q, which induces a fractional Ca^2+^ influx (Fig 3H and 3I), robustly triggers ADBE after high K^+^ stimulation (S7 Fig). However, high K^+^-induced bulk cisternae are significantly reduced in 4% FweE79Q-rescued larvae (Fig 4G, 4H and 4K). Notably, the number of high K^+^-induced bulk cisternae between 4% FweE79Q-rescued and *fwe* mutant larvae is comparable (*fwe* mutant larvae, 1.06 ± 0.4, *n* = 22, versus 4% FweE79Q-rescued larvae, 1.66 ± 0.42, *n* = 26, [Student’s *t* test, *p* = 0.31]). Similar to loss of *fwe*, there is an increase in the level of endocytic intermediates formed around the periactive zone in 4% FweE79Q-rescued boutons after high K^+^ stimulation when compared to 4% Fwe-rescued boutons (Fig 4L), thus supporting an important role of Ca^2+^ influx via Fwe in ADBE. At rest, the total SV membrane area per bouton area is also comparable between 4% Fwe- and 4% FweE79Q-rescued boutons (Fig 4M). Therefore, both expression conditions yield equal SV membranes available for SV exocytosis. These data suggest that Fwe triggers ADBE mainly through fluxing Ca^2+^. Furthermore, after chlorpromazine treatment, bulk membrane invaginations are less abundant in 4% FweE79Q-rescued boutons when compared to 4% Fwe-rescued boutons, also documenting a role of Fwe-derived Ca^2+^ influx in chlorpromazine-induced bulk membrane invagination (Fig 4N–4P).

If a suboptimal Ca^2+^ level in the presynaptic terminals results in impaired ADBE phenotype in 4% FweE79Q-rescued larvae, then we expected that increasing overall intracellular Ca^2+^ concentrations either via the remaining channel activity of FweE79Q or the other Ca^2+^ channels during stimulation might compensate for this low intracellular Ca^2+^ and rescue the defective ADBE. We therefore raised the Ca^2+^ concentration from 2 mM to 5 mM in our 90 mM K^+^ stimulation solution. When we applied a 10-min 90 mM K^+^/5 mM Ca^2+^ stimulation to 4% FweE79Q-rescued boutons, the bulk cisternae number is significantly rescued (Fig 4I and 4K). Similarly, 5mM Ca^2+^ also rescues the ADBE deficit associated with *fwe* mutants (Fig 4J and 4K). In contrast, this treatment does not increase the number of bulk cisternae further in 4% or 10% Fwe-rescued larvae when compared to the 10-min 90 mM K^+^/2 mM Ca^2+^ stimulation condition (Fig 4C, 4F and 4K). Hence, this rescue effect might be due to increased Ca^2+^ levels rather than enhanced ADBE in the presynaptic compartments. These data further support the role of Ca^2+^ influx via Fwe in triggering ADBE during intense activity stimulation.

### Lanthanum (La^3+^) blocks Fwe-mediated Ca^2+^ influx and impedes ADBE

**Fig 5:**
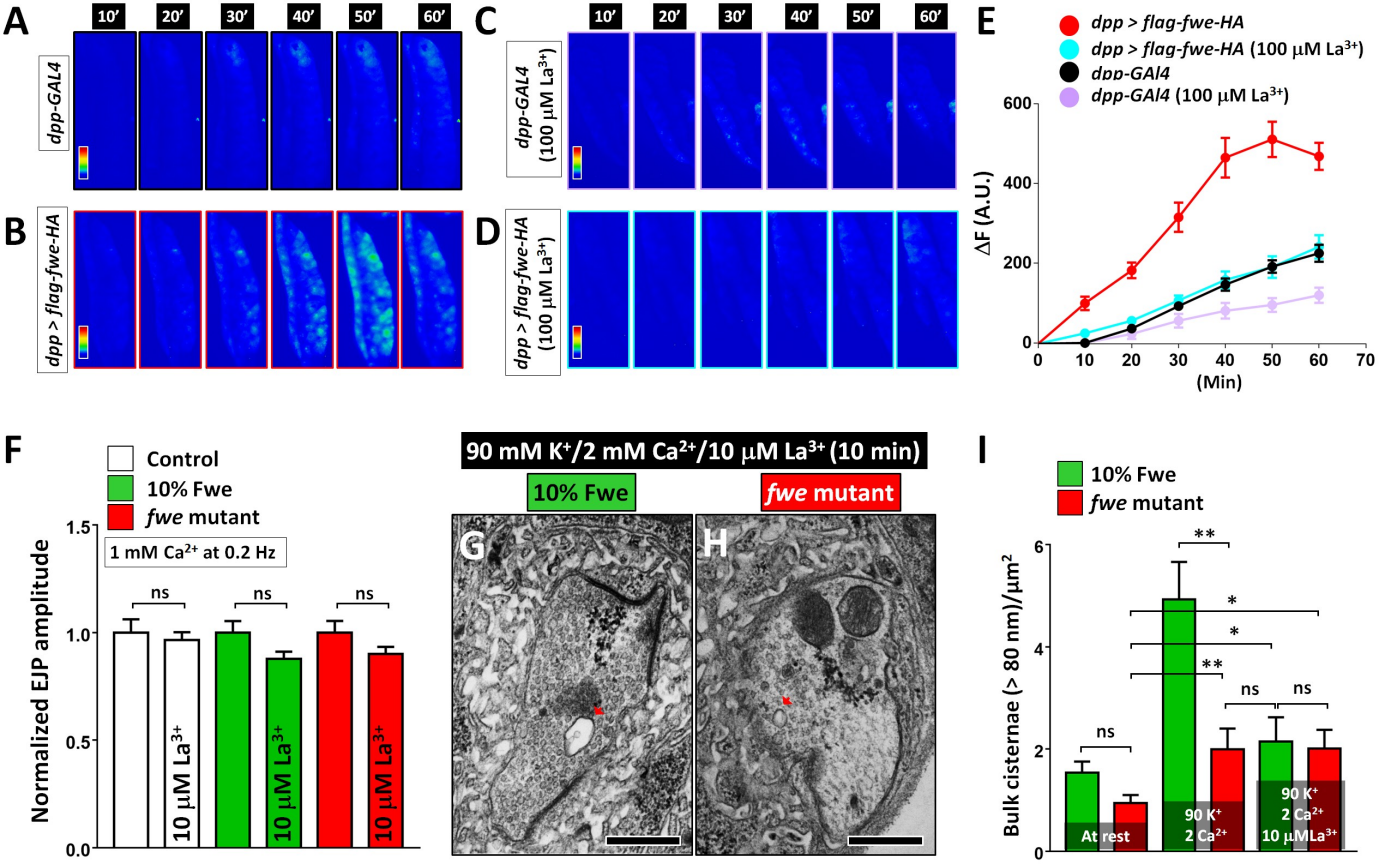
La^3+^ blocks Fwe-mediated Ca^2+^ uptake in salivary gland cells and impedes Activity-Dependent Bulk Endocytosis (ADBE) in high K^+^ stimulation. (A–D) Pseudocolored images of the third instar salivary gland cells are shown. The cells carrying *dpp-GAL4* driver alone (A, C) or *dpp>flag-fwe-HA* (B, D) were incubated in a solution of 4 μM Fluo 4-AM/100 μM Ca^2+^ (A, B) or 4 μM Fluo 4-AM/100 μM Ca^2+^/100 μM La^3+^ (C, D). (E) The time-course increase in the absolute unit (A.U.) of Fluo 4-AM fluorescence. The Ca^2+^ influx via Fwe in the salivary gland cells is blocked by La^3+^ treatment. Salivary glands (*dpp-GAL4*, *n* = 8; *dpp-GAL4* [100 μM La^3+^], *n* = 8; *dpp>flag-fwe-HA*, *n* = 11; and *dpp>flag-fwe-HA* [100 μM La^3+^], *n* = 9) derived from 5 larvae for each genotype were analyzed. (F) Larval fillets of *FRT80B* controls, 10% Fwe-rescued animals, and *fwe* mutants were incubated with 10 μM La^3+^-containing hemolymph-like (HL)-3 solution for 10 min, followed by 0.2 Hz stimulation in 1 mM Ca^2+^/10 μM La^3+^ HL-3 solution. Exocytosis elicited at 0.2 Hz is not affected by treatment with 10 μM La^3+^. A3 muscles 6 and 7 (control, *n* = 6; control [10 μM La^3+^], *n* = 7; 10% Fwe, *n* = 8; 10% Fwe [10 μM La^3+^], *n* = 8; *fwe* mutant, *n* = 7; and *fwe* mutant [10 μM La^3+^], *n* = 18) for each genotype were recorded. The values shown are normalized to the average value of untreated controls. (G–H) Transmission electron microscopy (TEM) images of neuromuscular junction (NMJ) boutons were obtained from 10% Fwe-rescued larvae (G) and *fwe* mutant larvae (H). Larvae were treated with a solution of 5 mM K^+^/1 mM Ca^2+^/10 μM La^3+^ for 10 min, subjected to 10-min 90 mM K^+^/2 mM Ca^2+^/10 μM La^3+^ stimulation, and eventually processed by standard TEM procedure. Bulk cisternae larger than 80 nm are indicated by red arrows. A 10 μM La^3+^ solution suppresses high K^+^-induced bulk cisternae in 10% Fwe-rescued larvae but not *fwe* mutant larvae. (I) Data quantifications of the number of bulk cisternae per bouton area. The data for 10% Fwe-rescued larvae and *fwe* mutant larvae at rest and after 90 mM K^+^/2 mM Ca^2+^ stimulation are derived from Figs 2G and 4K. Type Ib boutons (at rest: 10% Fwe, *n* = 17; and *fwe* mutant, *n* = 31. 10-min 90 mM K^+^/2 mM Ca^2+^ stimulation: 10% Fwe, *n* = 27; and *fwe* mutant, *n* = 22. 10-min 90 mM K^+^/2 mM Ca^2+^/10 μM La^3+^ stimulation: 10% Fwe, *n* = 38; and *fwe* mutant, *n* = 36.) derived from ≥3 larvae for each genotype were analyzed. A Student’s *t* test was used for statistical analysis. *p*-Value: ns, not significant; *, *p* < 0.05; **, *p* < 0.01. Error bars indicate the standard error of the mean. Scale bar: 500 nm in G–H. Images in A–D were taken at the same scale. The underlying data can be found in S1 Data.

If Ca^2+^ influx via Fwe triggers ADBE, one would anticipate that reducing channel activity will abolish ADBE. La^3+^ is a potent blocker of some Ca^2+^-permeable channels [39,40]. It may therefore inhibit the Fwe channel activity. We previously showed that heterologous expression of Fwe results in Ca^2+^ uptake by *Drosophila* salivary gland cells [24]. To determine the effect of La^3+^ on the Ca^2+^ conductance of Fwe, we applied 100 μM La^3+^ to the glands that carry a *GAL4* driver only or overexpress Fwe and performed Ca^2+^ imaging. The cells were loaded with Fluo-4 AM Ca^2+^ indicator and bathed in 100 μM extracellular Ca^2+^ solution. Fwe-overexpressing cells display a slow but significant Ca^2+^ uptake over a 1-h period when compared to controls (Fig 5A, 5B and 5E), consistent with our previous observations [24]. However, application of 100 μM La^3+^ solution nearly abolishes the Ca^2+^ influx mediated by Fwe (Fig 5D–5E), although a mild suppression was observed in control cells as well (Fig 5C and 5E). These results indicate that, similar to other Ca^2+^-permeable channels, the channel pore region of Fwe has a high affinity for La^3+^ and is blocked by La^3+^.

Next, we assessed the impact of 100 μM La^3+^ on ADBE. Since La^3+^ impedes Ca^2+^ permeability of VGCCs [39,40], and VGCC-triggered exocytosis is essential for ADBE initiation [7,26], we first tested whether treatment with100 μM La^3+^ solution affects SV exocytosis. At 0.2 Hz in 1 mM Ca^2+^, 100 μM La^3+^ reduces the amplitude of excitatory junction potentials (EJPs) by ~60%–80% when compared to untreated ones, indicating that La^3+^ blocks a Ca^2+^ influx mediated by VGCCs. In contrast, 10 μM La^3+^ does not significantly influence 0.2 Hz-elicited EJPs in controls (Fig 5F, white column). As this low La^3+^ concentration might not block the Fwe-derived Ca^2+^ influx effectively, we used 10% Fwe-rescued larvae, which are more sensitive to 10 μM La^3+^ (Fig 5B). Under this expression condition, the application of 10 μM La^3+^ does not affect the EJP responses at 0.2 Hz (Fig 5F, green column) but largely reduces ADBE upon high K^+^ stimulation (Fig 5G and 5I). Notably, the number of bulk cisternae induced under La^3+^ treatment is almost identical to that observed in *fwe* mutants after high K^+^-stimulation (Fig 5I), suggesting that La^3+^ suppresses ADBE by selectively inhibiting the channel activity of Fwe. In support of this, in *fwe* mutants, 10 μM La^3+^ does not alter the 0.2 Hz-evoked EJP amplitude (Fig 5F, red column) or the level of high K^+^-induced bulk cisternae (Fig 5H and 5I). Overall, these data indicate a role of Fwe in Ca^2+^-mediated ADBE. In summary, Fwe governs two major modes of SV endocytosis to permit consecutive rounds of exocytosis of neurotransmitters following distinct activity stimuli.

### Rodent Fwe isoform 2 is associated with SVs and can substitute the endocytic functions of *Drosophila* Fwe

**Fig 6:**
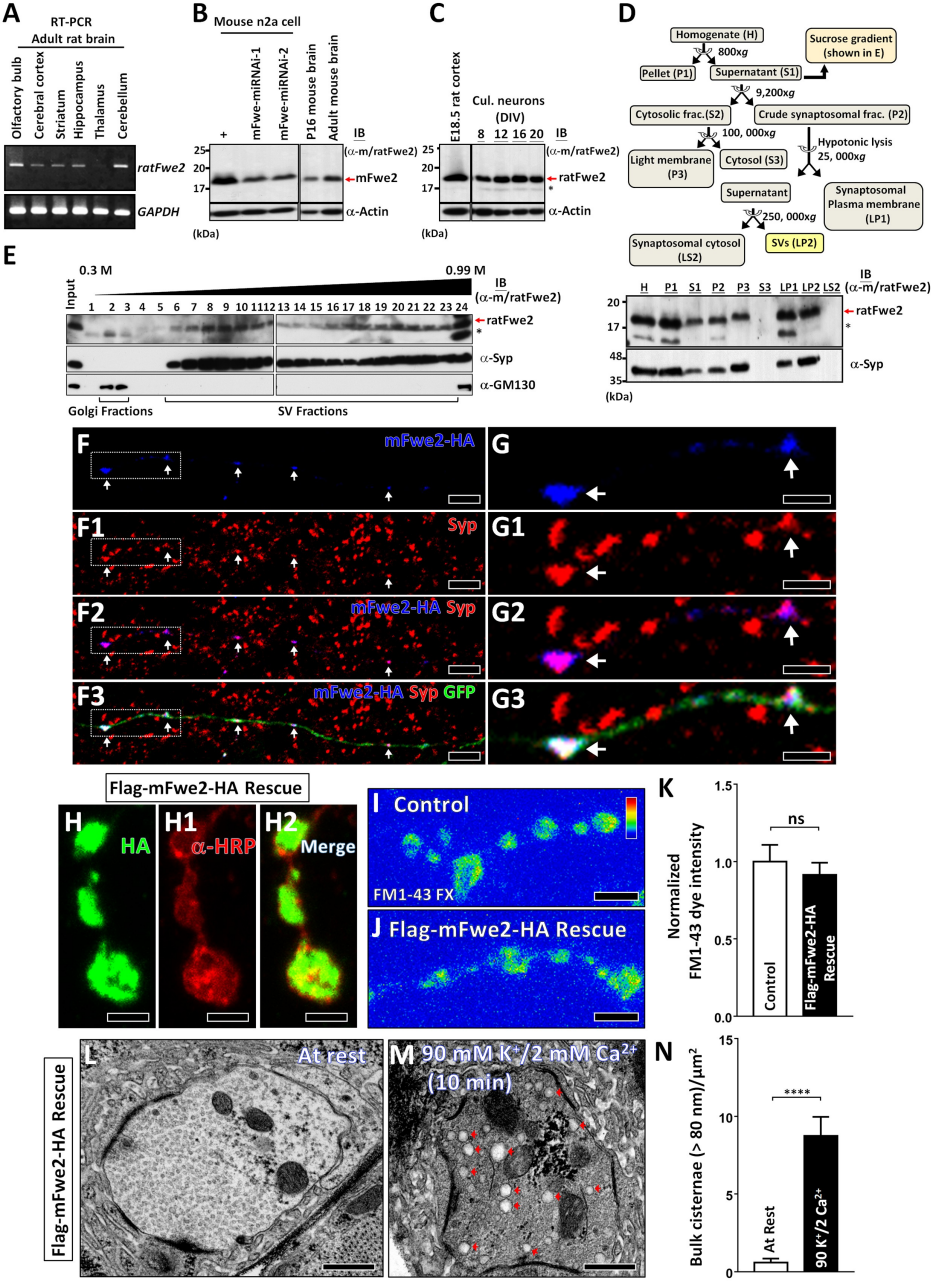
Rodent Fwe isoform 2 is localized on the Synaptic Vesicles (SVs) and triggers Clathrin-Mediated Endocytosis (CME) and Activity-Dependent Bulk Endocytosis (ADBE) at the *Drosophila* Neuromuscular Junction (NMJ). (A) In semiquantitative reverse-transcription PCR (RT-PCR) assays, the expression of *ratFwe2* mRNA was detected in distinct regions of adult rat brain. The PCR products of *ratFwe2* cDNA were sequenced to validate the identity of *ratFwe2* mRNA. The expression of *GAPDH* mRNA was used as the internal control. (B) In immunoblots with α-m/ratFwe2, mFwe2 was detected in the lysates of mouse neuroblastoma Neuro 2a (n2a) cells. This blotting signal is further reduced by expressing either one of two different mFwe-microRNAi (miRNAi). This isoform is also present in postnatal day 16 and adult mouse brains. Actin was used as the loading control. (C) In the immunoblots with α-m/ratFwe2, ratFwe2 was detected in the embryonic rat brain as well as in different days in vitro (DIV) cultured rat hippocampal neurons. Actin was used as the loading control. (D) The subcellular fractions were obtained from adult rat brain extracts using a series of centrifugations. ratFwe2 was found in the synaptosomal plasma membrane (lysate pellet 1 [LP1]) and SV fractions (lysate pellet 2 [LP2]) but was not detected in the cytosolic fraction (lysate supernatant 2 [LS2]). * indicates a degraded product of ratFwe2 or nonspecific antibody binding. (E) The subcellular fractions of adult rat brain were separated by 0.3–0.99 M sucrose gradients. ratFwe2 is present in the SV fractions containing Synaptophysin (Syp). Immunoblotting for GM130, a Golgi protein, labels Golgi fractions. (F–G) Confocal Z-projection images of DIV14 cultured rat hippocampal neurons stained with α-HA (blue), α-Syp (red), and α-GFP (green) were captured from neurons transfected with *pSpCas9(BB)-m/ratFwe-gRNA-2A-GFP-2A-mFwe2-HA* plasmid. White arrows indicate the presynaptic terminals. The enlarged images for white dashed boxes in F are shown in G. Axons are outlined by α-GFP staining. mFwe2-HA proteins are largely colocalized with Syp in the presynaptic terminals. (H–H2) Confocal Z-projection images of *Drosophila* NMJ boutons stained with α-HRP (red) and α-HA (green) were obtained from *fwe* mutants expressing Flag-mFwe2-HA (*nSyb(w) > flag-mFwe2-HA* in *fwe*^*DB25*^*/fwe*^*DB56*^). (I–J) Confocal Z-projection images of *Drosophila* NMJ boutons labeled with fixable FM1-43 dye were obtained from *FRT80B* control larvae (I) and Flag-mFwe2-HA-rescued larvae (J). CME was elicited by 1-min 90 mM K^+^/0.5 mM Ca^2+^ stimulation in the presence of 4 μM fixable FM1-43 dye. (K) Data quantifications for the FM1-43 dye intensity within the type Ib boutons shown in I–J. The FM1-43 dye fluorescence intensities were measured, and the values are normalized to the average value of controls. The neuronal expression of Flag-mFwe2-HA rescues the FM1-43 dye uptake defect in *fwe* mutants. Type Ib boutons of A2 muscles 6/7 were counted, and NMJs (control, *n* = 7; and Flag-mFwe2-HA rescue, *n* = 8) derived from 4 larvae were analyzed. (L–N) Transmission electron microscopy (TEM) images of NMJ boutons were captured from Flag-mFwe2-HA-rescued larvae. Samples were fixed under resting condition (10-min incubation in 5 mM K^+^/0 mM Ca^2+^ solution, L) or after 10-min 90 mM K^+^/2 mM Ca^2+^ stimulation (M). Data quantifications of the number of bulk cisternae per bouton area are shown in N. Type Ib boutons (at rest, *n* = 10; and 10-min 90 mM K^+^/2 mM Ca^2+^, *n* = 20) from 3 larvae for each condition were analyzed. A Student’s *t* test was used for statistical analysis. *p*-Value: ns, not significant; ****, *p* < 0.001. Error bars indicate the standard error of the mean. Scale bar: 2 μm (G–G3); 5 μm (F–F3, H–H2); 10 μm (I–J); 500 nm (L–M). The underlying data can be found in S1 Data.

Fwe homologs are found in most eukaryotes [24], but their role in SV endocytosis in vertebrates has not been established. The *mouse Fwe* (*mFwe*) gene can generate at least six alternative mRNA splicing isoforms [41], producing five different mFwe isoforms (S8 Fig). The mFwe isoform 2 (mFwe2) is the most similar to *Drosophila* Fwe. Moreover, the mFwe2 and rat Fwe isoform 2 (ratFwe2) share ~99% amino acid identity (170/172). In adult rat brain, *ratFwe2* mRNA is widely expressed (Fig 6A). In the lysates of mouse neuroblastoma Neuro 2a (n2a) cells, our antisera against the C-termini of both mFwe2 and ratFwe2 (α-m/ratFwe2) recognize a ~18 kDa protein band, corresponding to the predicted molecular weight of mFwe2 (Fig 6B). This signal is significantly decreased when *mFwe2* is knocked down by *mFwe-microRNAi (miRNAi)* (Fig 6B), showing antibody specificity. mFwe2 is expressed in postnatal as well as adult mouse brains (Fig 6B). Similarly, ratFwe2 was detected in rat brain and cultured rat hippocampal neurons (Fig 6C).

To determine if ratFwe2 is enriched in SVs, we purified SVs from adult rat brain using a series of centrifugations [42]. As shown in Fig 6D, ratFwe2 was specifically detected in the SV (Lysate pellet 2 [LP2]) fraction, marked by the presence of Synaptophysin (Syp), an abundant SV protein. ratFwe2 is also present in the SV fractions of adult rat brain separated with sucrose gradients (Fig 6E). To assess the subcellular localization of ratFwe2, we performed immunostaining in cultured neurons. Although staining with our antisera can visualize the expression of ratFwe2 in the cell bodies (S9B Fig), we failed to obtain specific staining in the presynaptic terminals. We therefore expressed HA-tagged mFwe2 in *ratFwe* knockout neurons (see below for details) and determined the SV localization of mFwe2-HA using α-HA staining. As shown in Fig 6F and 6G, mFwe2-HA protein is enriched in the presynaptic terminals and largely colocalized with Syp. The biochemical data combined with the *in vivo* localization data provide compelling evidence that ratFwe2 is associated with SV proteins, similar to *Drosophila* Fwe.

To determine whether rodent Fwe2 functions equivalently to *Drosophila* Fwe, we expressed *UAS-flag-mFwe2-HA* transgene in *fwe* mutants using *nSyb(w)-GAL4*. Overexpressed mFwe2 is localized to SVs in the boutons (Fig 6H–6H2) and rescues the FM1-43 dye uptake defect (Fig 6I–6K), as well as the reduced number of SVs (Fig 6L) in *fwe* mutants, showing that mFwe2 can promote CME in flies. Furthermore, the expression of mFwe2 corrects the ADBE deficit caused by loss of *fwe* (Fig 6M and 6N). Hence, mFwe2 promotes ADBE as well. We also observed that the early lethality of *fwe* mutant larvae is rescued by mFwe2 expression. Our results therefore suggest a conserved role of Fwe in SV endocytosis in mammals.

### The endocytic roles of Fwe are conserved at mammalian central synapses

To verify the role of ratFwe2 in SV endocytosis, we knocked out *ratFwe* in cultured rat hippocampal neurons using CRISPR/Cas9 technology [43]. We designed a specific guide RNA (gRNA; m/ratFwe-gRNA) that targets the first intron/second exon junction of both *mFwe* and *ratFwe* genes. To estimate the knockout efficiency, we transfected the gRNA construct into mouse neuroblastoma n2a cells and established a *mFwe* knockout n2a cell line. While mFwe2 was detected in normal n2a cells, it is lost in *mFwe* knockout n2a cells (S9A Fig). At 14 days in vitro (DIV), ratFwe2 is present in the Golgi apparatus of the cultured rat hippocampal neurons (S9B–S9B3 Fig), consistent with the fact that ratFwe2 is an SV protein sorted from the Golgi. In neurons expressing Cas9 and m/ratFwe-gRNA, the expression of ratFwe2 in the Golgi apparatus is significantly diminished (S9C and S9D Fig). Thus, this gRNA can also efficiently remove ratFwe2 in cultured neurons when Cas9 is present.

**Fig 7:**
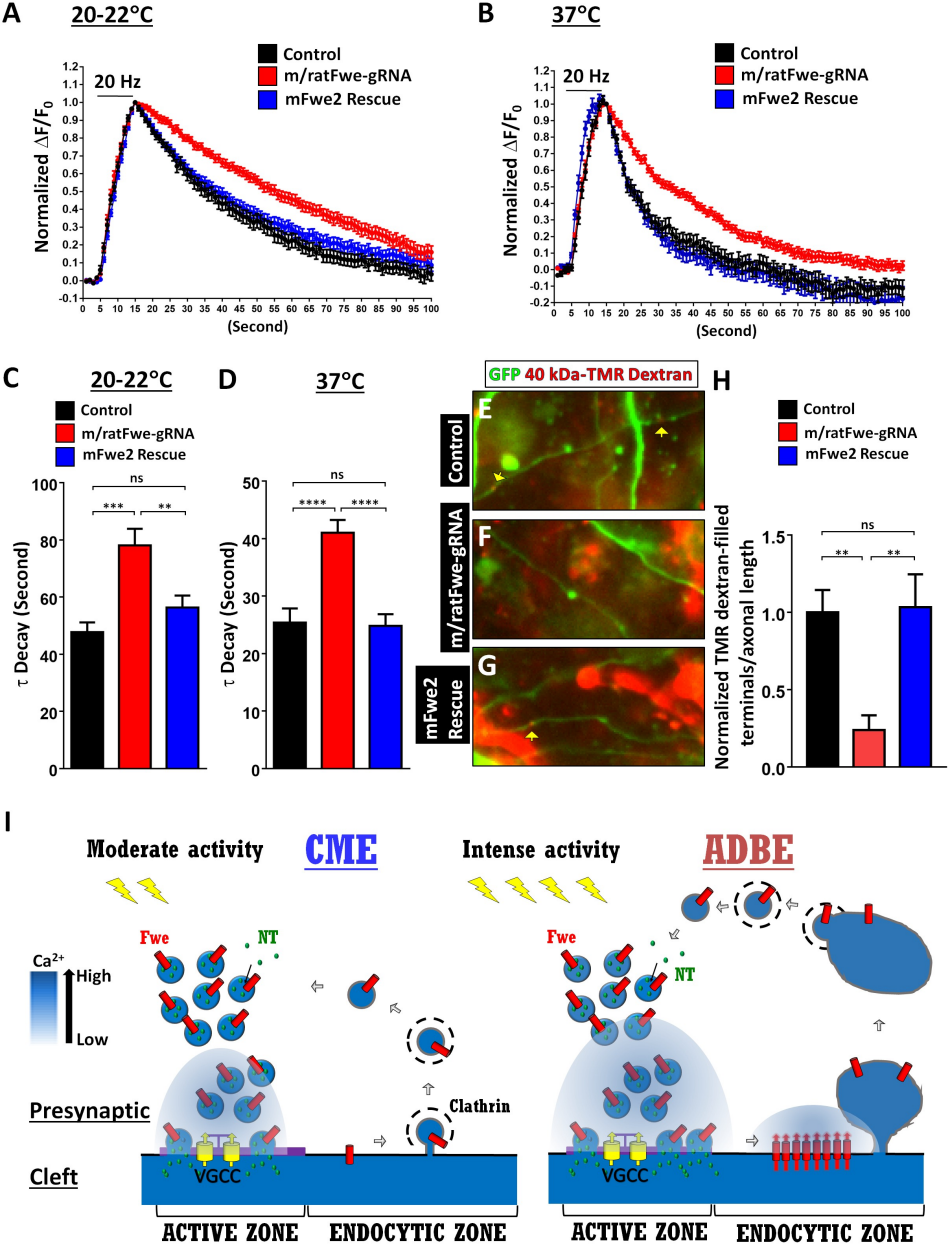
Knockout of *ratFwe2* impairs Clathrin-Mediated Endocytosis (CME) and Activity-Dependent Bulk Endocytosis (ADBE) in cultured rat hippocampal neurons. (A–D) The time-course traces for Synaptophysin-pHluorin (SypHy) fluorescence in the presynaptic terminals of DIV13–15 cultured rat hippocampal neurons. DIV7 neurons were transfected with *pSpCas9(BB)-2A-tagRFP* and *pCMV-SyphyA4* (black line), *pSpCas9(BB)-m/ratFwe-gRNA-2A-tagRFP* and *pCMV-SyphyA4* (red line), or *pSpCas9(BB)-m/ratFwe-gRNA-2A-tagRFP-2A-mFwe2-HA* and *pCMV-SyphyA4* (blue line). They were subjected to a train of 200 action potentials evoked at 20 Hz. The SypHy images were captured at room temperatures (20–22°C) and physiological temperatures (37°C). *ratFwe* knockout neurons display slow SypHy retrieval via synaptic vesicle (SV) endocytosis when compared to controls and mFwe2-rescued neurons. The average retrieval rates (τ) of SypHy are shown in C–D. Presynaptic terminals (20–22°C: control, *n* = 31; m/ratFwe-gRNA, *n* = 40; and mFwe2 rescue, *n* = 51. 37°C: control, *n* = 21; m/ratFwe-gRNA, *n* = 30; and mFwe2 rescue, *n* = 27) labeled with both tagRFP and SypHy derived from ≥5 coverslip cultures were analyzed. (E–G) DIV13–15 cultured rat hippocampal neurons were imaged after a train stimulation with 1,600 action potentials evoked at 80 Hz in the presence of 40 kDa tetramethylrhodamine (TMR)-dextran dye (red). The neurons were transfected with *pSpCas9(BB)-2A-GFP* (control, E), *pSpCas9(BB)-m/ratFwe-gRNA-2A-GFP* (m/ratFwe-gRNA, F), or *pSpCas9(BB)-m/ratFwe-gRNA-2A-GFP-2A-mFwe2-HA* (mFwe2 rescue, G) plasmid. In the axons marked with green fluorescent protein (GFP) expression, the presynaptic terminals filled with dextran dye are indicated by yellow arrows. (H) The number of dextran-filled presynaptic terminals per axonal length was quantified. The values shown in H are normalized to the average value of controls. The dextran uptake via ADBE is severely impeded in *ratFwe* knockout neurons, which is normalized by mFwe2 expression. Axons (control, *n* = 14; m/ratFwe-gRNA, *n* = 14; and mFwe2 rescue, *n* = 12) derived from ≥3 coverslip cultures were analyzed. The images were taken at the same scale. *p*-Value: ns, not significant; **, *p* < 0.01; ***, *p* < 0.01; ****, *p* < 0.001. Error bars indicate the standard error of the mean. (I) A proposed model for different roles of Fwe in CME and ADBE. The underlying data can be found in S1 Data.

To assess the efficacy of CME, we elicited exocytosis of Synaptophysin-pHluorin (SypHy) [5] by delivering 200 action potentials at 20 Hz and monitored its retrieval via SV endocytosis. It has been documented that this mild stimulation paradigm prevalently induces CME [12,15,44]. In control neurons (Fig 7A, black line) bathed at room temperature, repeated exocytosis in response to 20-Hz stimuli increases SypHy fluorescence, followed by a gradual fluorescence decay caused by the reacidification of SVs formed via CME. However, in *ratFwe* knockout neurons (Fig 7A, red line), the decay rate of SypHy fluorescence is much slower (Fig 7A and 7C). To verify whether this defect is specific to loss of ratFwe2, we expressed mFwe2-HA in *ratfwe* knockout neurons. mFwe2-HA properly localizes in the Golgi (S9E Fig) as well as in SVs (Fig 6F and 6G). This protein further normalizes the slow SypHy fluorescence decay (Fig 7A and 7C, blue line). Recent studies have revealed distinct properties of SV endocytosis under physiological conditions [15,45,46]. We found similar results when these recordings were performed at physiological temperatures (Fig 7B and 7D). A slow decay of SypHy fluorescence is possibly due to either impaired CME or inefficient SV reacidification or both. To distinguish these hypotheses, we performed an acidic quenching assay [47,48]. As shown in S10 Fig, upon perfusion of an acidic buffer, the newly recycled SVs in both control and *ratFwe* knockout neurons are efficiently acidified. Hence, our data suggest that ratFwe2 promotes CME at mammalian central synapses.

To assess the role of ratFwe2 in ADBE, we performed a dextran dye uptake assay in the control and *ratFwe* knockout neurons. We triggered ADBE with a stimulation of 1,600 action potentials delivered at 80 Hz in the presence of 40 kDa tetramethylrhodamine (TMR)-dextran [15,49]. As shown in Fig 7E–7H, in the control axons marked with green fluorescent protein (GFP) expression, the presynaptic terminals filled with dextran dye (red puncta) were observed frequently. In contrast, removal of *ratFwe* significantly diminishes dextran dye uptake. This phenotype is specific to loss of *ratFwe2*, as the reintroduction of mFwe2-HA corrects this dye uptake defect. Hence, ratFwe2 is indispensable for ADBE. In summary, Fwe promotes CME and ADBE in mammalian neurons, thereby coupling exocytosis to two major modes of endocytosis.

## Discussion

A tight coupling of exocytosis and endocytosis is critical for supporting continuous exocytosis of neurotransmitters. CME and ADBE are well-characterized forms of SV endocytosis triggered by moderate and strong nerve stimuli, respectively. However, how they are coupled with exocytosis under distinct stimulation paradigms remains less explored. Based on the present data, we propose a model as shown in Fig 7I. When presynaptic terminals are mildly stimulated, SV release leads to neurotransmitter release and the transfer of Fwe channel from SVs to the periactive zone where CME and ADBE occur actively [7,26,38]. Our data suggest that this channel does not supply Ca^2+^ for CME to proceed. However, intense activity promotes Fwe to elevate presynaptic Ca^2+^ levels near endocytic zones where ADBE is subsequently triggered. Thus, Fwe exerts different activities and properties in response to different stimuli to couple exocytosis to different modes of endocytosis.

We previously concluded that Fwe-dependent Ca^2+^ influx triggers CME [24]. However, the current results suggest alternative explanations. First, the presynaptic Ca^2+^ concentrations elicited by moderate activity conditions, i.e., 1-min 90 mM K^+^/0.5 mM Ca^2+^ or 20-s 10–20 Hz electric stimulation, are not dependent on Fwe (Fig 3G and 3H). Second, expression of 4% FweE79Q, a condition that abolishes Ca^2+^ influx via Fwe (Fig 3F), rescues the CME defects associated with *fwe* mutants, including decreased FM1-43 dye uptake, a reduced number of SVs, and enlarged SVs (Fig 1). Third, raising the presynaptic Ca^2+^ level has no beneficial impact on the reduced number of SVs observed in *fwe* mutants (Fig 4J). These data are consistent with the observations that a Ca^2+^ influx dependent on VGCCs triggers CME at a mammalian synapse [18,23]. Hence, Fwe acts in parallel with or downstream to VGCC-mediated Ca^2+^ influx during CME.

ADBE is triggered by intracellular Ca^2+^ elevation, which has been assumed to be driven by VGCCs that are located at the active zones [18,26]. However, our data strongly support a role for Fwe as an important Ca^2+^ channel for ADBE. First, following exocytosis, Fwe is enriched at the periactive zone where ADBE predominates [7,24,26]. Second, Fwe selectively supplies Ca^2+^ to the presynaptic compartment during intense activity stimulation (Fig 3), which is highly correlated with the rapid formation of ADBE upon stimulation [8,50]. Third, 4% FweE79Q expression, which induces very subtle or no Ca^2+^ upon strong stimulation, fails to rescue the ADBE defect associated with loss of *fwe* (Figs 3 and 4). Fourth, treatment with a low concentration of La^3+^ solution that specifically blocks the Ca^2+^ conductance of Fwe significantly abolishes ADBE (Fig 5). Lastly, the role of Fwe-derived Ca^2+^ influx in the initiation of ADBE mimics the effect of Ca^2+^ on ADBE at the rat Calyx of Held [7]. As loss of *fwe* does not completely eliminate ADBE, our results do not exclude the possibility that VGCC may function in parallel with Fwe to promote ADBE following intense stimulation.

Interestingly, Ca^2+^ influx via Fwe does not control SV exocytosis during mild and intense stimulations (S3 Fig). How do VGCC and Fwe selectively regulate SV exocytosis and ADBE, respectively? One potential mechanism is that VGCC triggers a high, transient Ca^2+^ influx around the active zone that elicits SV exocytosis. In contrast, Fwe is activated at the periactive zone to create a spatially and temporally distinct Ca^2+^ microdomain. A selective failure to increase the presynaptic Ca^2+^ level during strong stimulation is evident upon loss of *fwe*. This pinpoints to an activity-dependent gating of the Fwe channel. Consistent with this finding, an increase in the level of Fwe in the plasma membrane does not lead to presynaptic Ca^2+^ elevation at the Calyx of Held when the presynaptic terminals are at rest or subject to mild stimulation [23]. However, we previously showed that, in *shi*^*ts*^ terminals, blocking CME results in the accumulation of the Fwe channel in the plasma membrane, elevating Ca^2+^ levels [24]. It is possible that Dynamin is also involved in regulating the channel activity of Fwe or that the effects other than Fwe accumulation associated with *shi*^*ts*^ mutants may affect intracellular Ca^2+^ handling [51,52]. Further investigation of how neuronal activity gates the channel function of Fwe should advance our knowledge on the activity-dependent exo–endo coupling.

Although a proteomic analysis did not identify ratFwe2 in SVs purified from rat brain [53], our biochemical analyses show that ratFwe2 is indeed associated with the membrane of SVs. Our data show that 4% of the total endogenous Fwe channels efficiently promotes CME and ADBE at the *Drosophila* NMJ. If a single SV needs at least one functional Fwe channel complex during exo–endo coupling, and one functional Fwe complex comprises at least four monomers, similar to VGCCs, transient receptor potential cation channel subfamily V members (TRPV) 5 and 6, and calcium release-activated channel (CRAC)/Orai1 [24,40,54,55], then we anticipate that each SV contains ~100 Fwe proteins (4 monomers × 25). This suggests that Fwe is highly abundant on the SVs. It is unlikely that many SVs do not have the Fwe, as a 25-fold reduction of the protein is enough to ensure functional integrity during repetitive neurotransmission. Finally, our results for the SypHy and dextran uptake assays at mammalian central synapses indicate the functional conservation of the Fwe channel in promoting different modes of SV retrieval. In summary, the Fwe-mediated exo–endo coupling seems to be of broad importance for sustained synaptic transmission across species.

## Materials and methods

Detailed protocols are available at protocols.io (http://dx.doi.org/10.17504/protocols.io.hgbb3sn).

### *Drosophila* strains and genetics

Most of the experiments used *y w; FRT80B* isogenized fly, which was used for the generation of the *fwe*^*DB25*^ and *fwe*^*DB56*^ mutations [56] as the controls. Larvae were reared in standard fly food or on grape juice agar covered with yeast paste at 22°C. The genotypes of flies used in the experiments are described below.

For GCaMP6f imaging and immunostaining, the genotypes that carry *vglut-lexA* and *lexAop2-GCaMP6f* are as follows:

- control larvae (*w*^*1118*^*; vglut-lexA*, *13XLexAop2-IVS-GCaMP6f*; *nSyb(w)-GAL4*, *fwe*^*DB25*^*/+*). *fwe mutants* (*w*^*1118*^*; vglut-lexA*, *13XLexAop2-IVS-GCaMP6f*; *nSyb(w)-GAL4*, *fwe*^*DB25*^*/fwe*^*DB56*^)
- 4% Fwe-rescued larvae (*w*^*1118*^*; vglut-lexA*, *13XLexAop2-IVS-GCaMP6f/UAS-flag-fwe-RB-HA-1-2*; *nSyb(w)-GAL4*, *fwe*^*DB25*^/ *fwe*^*DB56*^)
- 4% FweE79Q-rescued animals (*w*^*1118*^*; vglut-lexA*, *13XLexAop2-IVS-GCaMP6f/UAS-flag-fweE79Q-RB-HA-6-1*; *nSyb(w)-GAL4*, *fwe*^*DB25*^/ *UAS-flag-fweE79Q-RB-HA-8-1*, *fwe*^*DB56*^)

The genotypes that carry *nSyb-GAL4* and *UAS-GCaMP6f* are as follows:

- control larvae (*w*^*1118*^*; 20XUAS-IVS-GCaMP6f* /+; *nSyb-GAL4*, *fwe*^*DB25*^/+)
- *fwe mutants* (*w*^*1118*^*; 20XUAS-IVS-GCaMP6f* /+; *nSyb-GAL4*, *fwe*^*DB25*^/ *fwe*^*DB56*^)
- 50% Fwe-rescued larvae (*w*^*1118*^*; 20XUAS-IVS-GCaMP6f*/*UAS-flag-fwe-RB-HA-1-2*; *nSyb-GAL4*, *fwe*^*DB25*^/ *fwe*^*DB56*^)
- 50% FweE79Q-rescued larvae (*w*^*1118*^*; 20XUAS-IVS-GCaMP6f* /*UAS-flag-fweE79Q-RB-HA-6-1*; *nSyb-GAL4*, *fwe*^*DB25*^/*UAS-flag-fweE79Q-RB-HA-8-1*, *fwe*^*DB56*^)
- *dap160 mutant* (*w*^*1118*^*; 20XUAS-IVS-GCaMP6f*, *dap160*^Δ2^*/dap160*^Δ1^; *nSyb-GAL4*/*+*)
- mFwe2-rescued larvae (*w*^*1118*^*; 20XUAS-IVS-GCaMP6f*/*UAS-flag-mFwe2-HA-2-1*; *nSyb-GAL4*, *fwe*^*DB25*^/ *fwe*^*DB56*^)
- *vglut-lexA* (BDSC #60314). *13XLexAop2-IVS-GCaMP6f* (BDSC #44277)
- *20XUAS-IVS-GCaMP6f* (BDSC #52869)
- *nSyb-GAL4* (BDSC #51635)
- *dap160*^Δ1^ and *dap160*^Δ2^*[36]*

The following *UAS* transgenes were made in this study:

- *UAS-flag-fwe-RB-HA-1-2*, *UAS-flag-fweE79Q-RB-HA-6-1*, *UAS-flag-fweE79Q-RB-HA-8-1*, and *UAS-flag-mFwe2-HA-2-1*.

For electrophysiology, the following were used:

- control larvae (*y w; P{ry*^*+*^ *neoFRT}80B/ P{ry*^*+*^ *neoFRT}80B*)
- *fwe* mutant larvae (*w*^*1118*^; *fwe*^*DB25*^/ *fwe*^*DB56*^)
- 10% Fwe-rescued larvae (*w*^*1118*^; *elav-GAL4*/*UAS-flag-fwe-RB-HA-1-2*; *fwe*^*DB25*^/ *fwe*^*DB56*^)

For Cac-EGFP expression, the following were used:

- control larvae (*w*^*1118*^*; UAS-cac1-EGFP422A*/+; *nSyb-GAL4*/ *fwe*^*DB25*^)
- *fwe mutants* (*w*^*1118*^*; UAS-cac1-EGFP422A* /+; *nSyb-GAL4*, *fwe*^*DB25*^/ *fwe*^*DB56*^)
- *UAS-cac1-EGFP(422A)* (BDSC #8765)

Those used for Fluo-4 AM experiments in salivary glands are as follows:

- control larvae (*w*^*1118*^*; +/+; dpp-GAL4*/+)
- *fwe* overexpression (*w*^*1118*^*; UAS-flag-fwe-RB-HA-20-1/+; dpp-GAL4*/+)
- *dpp-GAL4* and *UAS-flag-fwe-RB-HA-20-1* [24]

Those used for TEM analysis, FM1-43 dye uptake assay, immunostaining of HA, and Fwe are as follows:

- control larvae (*y w; P{ry*^*+*^ *neoFRT}80B/ P{ry*^*+*^ *neoFRT}80B*). *fwe* mutant larvae (*w*^*1118*^; +/+; *fwe*^*DB25*^/ *fwe*^*DB56*^)
- Genomic HA-Fwe-rescued larvae (*genomic HA-fwe construct-17-1/+*; *fwe*^*DB25*^/*fwe*^*DB56*^)
- 50% Fwe-rescued larvae (*w*^*1118*^; *UAS-flag-fwe-RB-HA-1-2*/+; *nSyb-GAL4*, *fwe*^*DB25*^/ *fwe*^*DB56*^)
- 50% FweE79Q-rescued larvae (*w*^*1118*^*; UAS-flag-fweE79Q-RB-HA-6-1/+*; *nSyb-GAL4*, *fwe*^*DB25*^/*UAS-flag-fweE79Q-RB-HA-8-1*, *fwe*^*DB56*^)
- 10% Fwe-rescued larvae (*w*^*1118*^; *elav-GAL4*/*UAS-flag-fwe-RB-HA-1-2*; *fwe*^*DB25*^/ *fwe*^*DB56*^)
- 10% FweE79Q-rescued larvae (*w*^*1118*^*; elav-GAL4/UAS-flag-fweE79Q-RB-HA-6-1*; *fwe*^*DB25*^/*UAS-flag-fweE79Q-RB-HA-8-1*, *fwe*^*DB56*^)
- 4% Fwe-rescued larvae (*w*^*1118*^; *UAS-flag-fwe-RB-HA-1-2*/+; *nSyb(w)-GAL4*, *fwe*^*DB25*^/ *fwe*^*DB56*^)
- 4% FweE79Q-rescued animals (*w*^*1118*^*; UAS-flag-fweE79Q-RB-HA-6-1/+*; *nSyb(w)-GAL4*, *fwe*^*DB25*^/*UAS-flag-fweE79Q-RB-HA-8-1*, *fwe*^*DB56*^)
- *mFwe2*-recsued larvae (*w*^*1118*^*; UAS-flag-mFwe2-HA-2-1/+*; *nSyb(w)-GAL4*, *fwe*^*DB25*^/ *fwe*^*DB56*^)
- *elav-GAL4* (BDSC #8765)
- For generating *nSyb(w)-GAL4*, *nSyb(w)-GAL4* that weakly drives gene expression was recombined out from *nSyb-GAL4* line (BDSC #51635) that carries at least two *nSyb-GAL4* insertions.

### Molecular cloning

The *pCasper4-genomic HA-fwe* construct was constructed by inserting a HA sequence to the site after the translational start codon of *fwe*-*RB* in the context of the *pCasper4-genomic fwe* construct [24]. To obtain the *pUAST-flag-mFwe2-HA* construct, the *fwe-RB* fragment of the *pUAST-flag-fwe-RB-HA* construct [24] was replaced with the *mFwe2* coding region, which was amplified from total mRNA of the adult mouse brain. P-element-mediated transgenesis was achieved by the standard procedure. The introduction of these genomic *fwe* transgenes to the *fwe* mutant background rescues the early lethality associated with *fwe* mutants, demonstrating that fused tags do not affect normal functions of Fwe. To generate the *mFwe-miRNAi* constructs, the sequences of miRNAs were designed according to Invitrogen’s RNAi Designer. *mFwe-miRNAi-1* targets nucleotides 183–203 of the *mFwe2* coding sequence (forward oligomer: TGCTGAAGG CGTTCATGATCATCCACGTTTTGGCCACTGACTGACGTGGATGAATGAACGCCTT; reverse oligomer: CCTGAAGGCGTTCATTCATCCACGTCAGTCAGTGGCCAAAACGTGG ATGATCATGAACGCCTTC). *mFwe-miRNAi-2* targets nucleotides 236–256 of the *mFwe2* coding sequence (forward oligomer: TGCTGTTG CAAACTCCACAAACTGGCGTTTTGGC CACTGACTGACGCCAGTTTGGAGTTTGCAA; reverse oligomer: CCTGTTGCAAACTCC AAACTGGCGTCAGTCAGTGGCCAAAACGCCAGTTTGTGGAGTTTGCAAC). Synthetic oligomers were annealed and subcloned to pcDNA 6.2-GW/EmGFPmiR vector (Invitrogen). To generate the pSpCas9(BB)-based plasmids, pSpCas9(BB)-2A-tagRFP was constructed by replacing the GFP region of the pSpCas9(BB)-2A-GFP plasmid (addgene#48138) [43] with a TagRFP coding sequence. m/ratFwe-gRNA was designed to target the first intron/second exon junction of the *mFwe* and *ratFwe* (forward oligomer: CACCGTTTGAAGCCTGTGCCATCTC; reverse oligomer: TTTGCTCTACCGTGTCCGAAG TTTG). Annealed synthetic oligomers were placed into the *BbsI* site of pSpCas9(BB)-2A-GFP and pSpCas9(BB)-2A-tagRFP to obtain pSpCas9(BB)-m/ratFwe-gRNA-2A-GFP and pSpCas9(BB)-m/ratFwe-gRNA-2A-tagRFP constructs. pSpCas9(BB)-m/ratFwe-gRNA-2A-GFP-2A-mFwe2-HA and pSpCas9(BB)-m/ratFwe-gRNA-2A-tagRFP-2A-mFwe2-HA were generated by inserting the DNA fragment of *2A-mFwe2-HA*, which was amplified by PCR with the primers (forward primer: AAAAGCTTGGCAGTGGAGAGGGCAGAGGAAGTCTGCTAACATGCGGTGACGTCG AGGAGAATCCTGGCCCAAGCGGCTCGGGCGCC; reverse primer: CCCTCGAGTTA CGCGTAGTCGGGGAC), after GFP or tagRFP.

### Reverse-transcription PCR (RT-PCR)

Total RNA of different regions of the adult rat brain were extracted with TRIZOL reagent according to the manufacturer's instructions. Five μg of total RNA was mixed with oligo-dT primer in 20 μl of reverse transcription reaction solution. One μl of this mixture was used to amplify the cDNA of *ratFwe2* mRNA with specific primers (forward primer: GAAGATCTATGAGCGGCTCGGTCGCC; reverse primer: CGGAATTCTCACAGTTCCCCCTCGAATG). Twenty-five PCR cycles were used to allow exponential PCR amplification. The PCR products were sequenced to validate the identity of *ratFwe2* mRNA.

### Antibody generation

GST-fused polypeptides comprising seven tandem repeats of the C-terminus of *Drosophila* Fwe-PB isoform [24] were injected in guinea pigs to obtain GP100Y antisera. To generate α-m/ratFwe2 antisera (GP67), the DNA fragment encoding seven tandem repeats of ratFwe2 C-terminus (a.a. 140–172) was subcloned to pET28a plasmid. His-fused polypeptides were purified and then injected into guinea pigs. Antibody generation was assisted by LTK BioLaboratories (Taiwan). Specific antibodies were further purified by antigen-conjugated affinity columns.

### Immunohistochemistry

For immunostaining in fly NMJ boutons, larval fillets were dissected in ice-cold 1X PBS and fixed in 4% paraformaldehyde for 20 min at room temperature. The samples were permeabilized in 0.1% Triton X-100-containing 1X PBS solution for all staining, except staining with anti-Fwe (GP100Y) and anti-HA antibodies, which used 0.1% Tween-20-containing 1X PBS solution to prevent Fwe dissociation from the SVs. Primary antibody dilutions used mouse anti-Dlg (mAb 4F3) [57], 1:100 (Hybridoma bank) [58]; mouse anti-Brp (nc82), 1:100 (Hybridoma bank) [59]; rabbit anti-GFP, 1:500 (Invitrogen); mouse monoclonal anti-HA, 1:200 (Sigma); rabbit anti-HA, 1:200 (Sigma); rabbit Cy3 conjugated anti-HRP, 1:500 (Jackson ImmunoResearch); and guinea pig anti-Fwe-PB (GP100Y), 1:100. Secondary antibodies were diluted to 1:500 (Jackson ImmunoResearch and Invitrogen). For immunostaining of cultured rat hippocampal neurons, DIV14 neurons were fixed in 4% paraformaldehyde/4% sucrose for 10 min at room temperature and permeabilized and washed with 0.1% Tween-20-containing 1X PBS solution. To reduce nonspecific staining, GP67 antibodies were absorbed with paraformaldehyde-fixed n2a cells before staining. Primary antibody dilutions used rabbit anti-GFP, 1:500 (Invitrogen); guinea pig ant-m/ratFwe2 (GP67), 1:100; rabbit anti-GM130, 1:500 (Adcam); mouse anti-HA, 1:200 (Sigma); and mouse anti-SVP38, 1:200 (Sigma). Secondary antibodies were diluted to 1:500 (Jackson ImmunoResearch and Invitrogen). DAPI was used in the 1:2,000 dilution (Invitrogen). To compare the staining intensity of boutons among different genotypes, larval fillets used in the same graph were stained in the same Eppendorf tube. The images were captured using a Zeiss 780 confocal microscope, and the scan setup was fixed for the same experimental set. For data quantifications, single-plane confocal images were projected. The final staining intensity in boutons was calculated by subtracting the background fluorescence intensity in the surrounding muscles from the staining intensity in boutons. The staining intensities of all type Ib boutons from the same muscles 6 and 7 in one image were averaged to obtain each data value. Image processing was achieved using LSM Zen and Image J.

### Generation of mFwe knockout neuroblastoma n2a cells

Mouse neuroblastoma n2a cells were transfected with pSpCas9(BB)-m/ratFwe-gRNA-2A-GFP plasmid. GFP-positive cells were sorted out using flow cytometry, and the cells were plated in a 96-well plate in which each well included approximately one cell. After 3-wk culture, single cell-driven colonies were subjected to immunostaining and immunoblotting for mFwe2 to verify the knockout of mFwe2. One of the confirmed mFwe knockout neuroblastoma n2a cell lines was used in S9A Fig.

### Fractionation and western blotting

For western blotting of n2a cell lysates, the cells lysed with RIPA buffer (50 mM Tris-HCl [pH 8], 150 mM NaCl, 1% NP-40, 0.5% sodium deoxycholate, and 0.1% SDS) were boiled in 1X SDS sample buffer for 10 min. To prepare subcellular fractions of adult rat brain, one adult brain (~1 g) was homogenized in 5 ml 0.32 M sucrose buffer (320 mM sucrose, 1.5 mM MgCl_2_, 1 mM EGTA, and 10 mM HEPES [pH 7.5]) using a Teflon glass homogenizer. The homogenates (H) were centrifuged at 800 × g for 15 min at 4°C to yield pellets (P1) and supernatants (S1). S1 supernatants were centrifuged at 9,200 × g for 15 min at 4°C to obtain pellets (P2) and supernatants (S2), which were centrifuged at 100,000 × g for 2 h at 4°C to obtain fractions of cytosol (S3) and light membrane (P3). The pellets (P2) were then lysed in ice-cold Mini-Q water, followed by equilibration with 4 mM HEPES. After 30-min mixing at 4°C, the lysates were centrifuged at 25,000 × g for 20 min at 4°C to yield the crude synaptic vesicle fraction (LS1) and lysed synaptosomal membrane fraction (LP1). The LS1 fraction was further centrifuged at 100,000 × g to obtain crude synaptic vesicles (LP2) and the synaptosomal cytosol fraction (LS2). A discontinuous sucrose gradient from 0.3–0.99 M was prepared by gradually layering the different concentrations of sucrose. The S1 supernatants were loaded on sucrose gradient solution and centrifuged at 33,000 × g for 3 h at 4°C. Fractions were collected from low- to high-density sucrose. These fractions were boiled in 1X SDS sample buffer and subjected to SDS-PAGE and western blotting. The primary antibody dilutions used were as follows: guinea pig anti-m/ratFwe2 (GP67), 1:500; rabbit anti-SVP38, 1:1,000 (Sigma); rabbit anti-GM130, 1:5,000 (Abcam); mouse anti-Tubulin, 1: 10,000 (Sigma); and mouse anti-α-Actin, 1:10,000 (Sigma). Secondary HRP-conjugated antibodies were diluted to 1:5,000 (Jackson ImmunoResearch).

### FM1-43 dye uptake

To induce CME, the third instar larvae were dissected in 0 mM Ca^2+^ hemolymph-like (HL)-3 solution at room temperature (70 mM NaCl, 5 mM KCl, 10 mM MgCl_2_, 10 mM NaHCO_3_, 5 mM trehalose, 5 mM HEPES [pH 7.2], and 115 mM sucrose) [60] and subjected to 1-min 90 mM K^+^/0.5 mM Ca^2+^ stimulation (25 mM NaCl, 90 mM KCl, 10 mM MgCl_2_, 10 mM NaHCO_3_, 5 mM trehalose, 5 mM HEPES [pH 7.2], 30 mM sucrose, and 0.5 mM CaCl_2_) or 10-min 60 mM K^+^/1 mM Ca^2+^ stimulation (55 mM NaCl, 60 mM KCl, 10 mM MgCl_2_, 10 mM NaHCO_3_, 5 mM trehalose, 5 mM HEPES [pH 7.2], 30 mM sucrose, and 1 mM CaCl_2_) in the presence of 4 μM fixable FM1-43 (Invitrogen). Excess dye was extensively washed with 0 mM Ca^2+^ HL-3 solution for 10 min. Larval fillets were fixed in 4% paraformaldehyde for 10 min, washed, mounted, and imaged on a Zeiss 780 confocal microscope. The scan setup was fixed for all the sets of the experiments. For data quantifications, single-plane confocal images were projected, and the final FM1-43 dye intensity in the boutons was calculated by subtracting the dye fluorescence intensity in the surrounding muscles from the dye fluorescence intensity within the boutons. The dye fluorescence intensities of all type Ib boutons from the same muscles 6 and 7 were averaged to obtain each data value. For chlorpromazine treatment experiment, dissected larvae were incubated with 200 μM chlorpromazine (Sigma) in Schneider medium for 30 min. They were then stimulated with a solution of 90 mM K^+^/2 mM Ca^2+^/200 μM chlorpromazine (25 mM NaCl, 90 mM KCl, 10 mM MgCl_2_, 10 mM NaHCO_3_, 5 mM trehalose, 5 mM HEPES [pH 7.2], 30 mM sucrose, 2 mM CaCl_2_, and 200 μM chlorpromazine) in the presence of 4 μM fixable FM1-43 for 10 min. Bulk membranous invaginations were defined as the internalized structures labeled with high levels of FM1-43 dye. The areas of individual type Ib boutons and bulk membranous invaginations were measured using Image J. For FM1-43 dye loading/unloading assays, larvae were dissected in 0 mM Ca^2+^ HL-3 solution at room temperature and subjected to a stimulation of 90 mM K^+^/0.5 (or 2) mM Ca^2+^ HL-3 solution for 5 min to load boutons with the FM1-43 dye. Excess dye was removed by extensive washing with 0 mM Ca^2+^ HL-3 solution for 10 min. FM1-43 dye uptake by boutons was imaged to indicate “loading.” Subsequently, the dye loaded in SVs was unloaded by stimulation using 90 mM K^+^/0.5 (or 2) mM Ca^2+^ solution for 1 min. Released dye was removed by several washes with a 0 mM Ca^2+^ HL-3 solution. The remaining dye in boutons was imaged to indicate “unloading.” The final FM1-43 dye intensity in the boutons was calculated by subtracting the dye fluorescence intensity in the surrounding muscles from the dye fluorescence intensity within the boutons. The dye fluorescence intensities of at least ten type Ib boutons from the same muscles 6 and 7 were averaged to obtain each data value. The dye unloading efficiency was indicated as (F_load_-F_unload_)/F_load_. Images processing was achieved using Image J and LSM Zen.

### Ca^2+^ imaging

#### GCaMP6f imaging

The third instar larvae were dissected in 0 mM Ca^2+^ HL-3 at room temperature and incubated in 2 mM Ca^2+^/5 mM K^+^/7 mM glutamate solution (70 mM NaCl, 5 mM KCl, 10 mM MgCl_2_, 10 mM NaHCO_3_, 5 mM trehalose, 5 mM HEPES [pH 7.2], 115 mM sucrose, 2 mM CaCl_2_, and 7 mM monosodium glutamate). Glutamate treatment would desensitize postsynaptic glutamate receptors, thus reducing muscle contraction upon stimulation. GCaMP6f fluorescence was then measured to indicate the resting Ca^2+^ levels. To image GCaMP6f in high K^+^ stimulations, larval fillets were subsequently stimulated with 90 mM K^+^/2 mM Ca^2+^/7 mM glutamate solution. High K^+^ and Ca^2+^ lead to bulk Ca^2+^ influxes into the muscles and cause dramatic contractions. The boutons were manually focused and simultaneously imaged in the 6th and 10th min every 1 s. After 10-min stimulation, larval fillets were rinsed with 2 mM Ca^2+^/5 mM K^+^/7 mM glutamate solution and imaged again. Similarly, boutons subjected to 1-min 90 mM K^+^/0.5 mM Ca^2+^/7 mM glutamate stimulation were manually focused and imaged between 30–60 s every 1 s. All images were captured from muscles 6 and 7 of abdominal segment 3. Each larva was only used for one recording. The images of clearly focused boutons were further used for data quantifications. The GCaMP6f fluorescence intensities in type Ib boutons and the surrounding muscles (served as the fluorescence background) were measured. Final GCaMP6f fluorescence intensity was calculated by subtracting the background fluorescence intensity in the surrounding muscles from the GCaMP6f fluorescence intensity in the boutons. The GCaMP6f fluorescence intensities of at least ten type Ib boutons from the same muscles 6 and 7 at a given time period were averaged to obtain each data value. For electric stimulation, the larval axonal bundle was aspirated and delivered with 10–40 Hz stimulations via a glass capillary electrode. The stimulus was fixed at 5 mV and 0.5 ms duration by pClamp 10.6 software (Axon Instruments). The images were captured every 2 s using MetaMorph software and an ANDOR iXon3 897 camera. All images were captured from muscles 6 and 7 of abdominal segment 3. Each larva was only used for one recording. The GCaMP6f fluorescence intensities in type Ib boutons and the surrounding muscles (which served as the fluorescence background) were measured. The final GCaMP6f fluorescence intensity was calculated by subtracting the background fluorescence intensity in the surrounding muscles from the GCaMP6f fluorescence intensity in the boutons. The GCaMP6f fluorescence intensities of at least five type Ib boutons from the same muscles 6 and 7 at a given time period were averaged to obtain each data value. Images processing was achieved using Image J and LSM Zen.

#### Ca^2+^ imaging in salivary glands

Briefly, the gland cells of the third instar larvae were dissected in 0 mM Ca^2+^ HL-3 solution. The cells were subjected to loading of 4 μM Fluo-4 AM (Invitrogen) in 100 μM Ca^2+^ solution. For La^3+^ treatment, the cells were incubated in 100 μM LaCl_3_/100 μM Ca^2+^ solution. The images were captured every 10 min during dye loading using MetaMorph software and an ANDOR iXon 897 camera. The fluorescence intensities in the salivary gland cells and surrounding cover slips were measured. The final fluorescence value was calculated by subtracting the fluorescence intensity in the gland cells from the dye fluorescence intensity in the coverslips. The fluorescence intensity of one salivary gland was used for each data value. Image processing was achieved using Image J and LSM Zen.

### Electrophysiology

The third instar larvae were dissected in 0 mM Ca^2+^ HL-3 at room temperature and then bathed in 1 mM Ca^2+^ HL-3 solution for 5–10 min before the recording. The mean value of the resistance of the recording electrode was ~40 MΩ when the electrode was filled with a 3M KCl solution. All recordings were obtained from muscle 6 of abdominal segment 3. Each larva was only used for one recording. Recordings from the muscles that hold resting membrane potentials at less than −60 mV were used for further data quantifications. EJPs were evoked by stimulating the axonal bundle via a glass capillary electrode with an internal diameter of ~10–15 μm (Harvard Apparatus Glass Capillaries GC120F-15) at 0.2 Hz. Stimulus pulses were fixed at 0.5 ms duration (pClamp 10.6 software, Axon Instruments). To obtain maximal EJP amplitude, 3–5 mV electric stimuli were applied. EJPs were amplified with an Axoclamp 900A amplifier (Axon Instruments, Foster City, California) under bridge mode and filtered at 10 kHz. EJPs were analyzed by pClamp 10.6 software (Axon Instruments). For the EJP amplitude at 0.2 Hz, the mean of the EJP amplitude was averaged from the amplitudes of 80 EJPs in one consecutive recording.

### TEM

Larval fillets were dissected in 0 mM Ca^2+^ HL-3 medium at room temperature. To trigger CME, samples were stimulated with 90 mM K^+^/0.5 mM Ca^2+^ HL-3 solution for 1 min or 60 mM K^+^/1 mM Ca^2+^ HL-3 solution for 10 min. To induce ADBE, larval fillets were subjected to stimulation of a 90 mM K^+^/2 mM or 5 mM Ca^2+^ HL-3 solution in the presence or lack of 10 μM La^3+^ for 10 min. Subsequently, the samples were fixed for 12 h in 4% paraformaldehyde/1% glutaraldehyde/0.1 M cacodylic acid (pH 7.2) solution and then rinsed with 0.1 M cacodylic acid (pH 7.2) solution. They were subsequently fixed in 1% OsO4/0.1 M cacodylic acid solution at room temperature for 3 h. The samples were subjected to a series of dehydration from 30% to 100% ethanol. After 100% ethanol dehydration, the samples were incubated in propylene, a mixture of propylene and resin, and pure resin. Lastly, they were embedded in 100% resin. The images of type Ib boutons were captured using Tecnai G2 Spirit TWIN (FEI Company) and a Gatan CCD Camera (794.10.BP2 MultiScan) at ≥4,400 × magnifications. The size of the SVs and the bulk cisternae and the area of type Ib boutons were measured using Image J. We identified type Ib boutons by multiple layers of subsynaptic reticulum. The radius of the bulk cisternae was calculated from A(area) = πr^2^. Isolated membranous structures larger than 80 nm in diameter were defined as bulk cisternae.

### SypHy imaging and dextran uptake assays

For SypHy imaging, DIV7 cultured rat hippocampal neurons were transfected with pSpCas9(BB) and pCMV-SyphyA4 (addgene#24478) plasmids [5]. DIV13–15 neurons were bathed in the imaging buffer in a chamber (Warner instruments RC-25F) with two parallel platinum wires separated by 5 mm. The imaging buffer consisted of 136 mM NaCl, 2.5 mM KCl, 2 mM CaCl_2_, 1.3 mM MgCl_2_, 10 mM glucose, 10 mM HEPES (pH 7.4), 10 μM CNQX, and 50 μM AP-5. SV exocytosis was elicited with a train of 200 action potentials delivered with a 20-Hz electric field stimulation (50 mA, 1-ms pulse width). Single images were captured every 1 s using MetaMorph software and an ANDOR iXon 897 camera. The experiments were performed at either room or physiological temperatures controlled by a Warner temperature controller (TC-344B). For SV reacidification experiments, the imaging chamber was perfused with the imaging buffer (136 mM NaCl, 2.5 mM KCl, 2 mM CaCl_2_, 1.3 mM MgCl_2_, 10 mM glucose, and 10 mM HEPES [pH 7.4], 10 μM CNQX, and 50 μM AP-5) before perfusion with an acidic buffer (136 mM NaCl, 2.5 mM KCl, 2 mM CaCl_2_, 1.3 mM MgCl_2_, 10 mM glucose, and 10 mM 2-[N-morpholino] ethane sulphonic acid [pH 5.5], 10 μM CNQX, and 50 μM AP-5), which was prepared by replacing HEPES in the imaging buffer with 2-[N-morpholino] ethane sulphonic acid [47,48]. Next, the imaging buffer was perfused to allow surface SypHy to be fluorescent. Experimental temperatures were maintained at physiological temperatures. The final SypHy fluorescence intensities in the presynaptic terminals were calculated by subtracting the background fluorescence intensity on the surrounding coverslip from the SypHy fluorescence intensity within presynaptic terminals. Each data value was obtained from a single terminal. For 40 kDa TMR-dextran uptake assays, DIV13–15 neurons transfected with pSpCas9(BB) plasmids were stimulated by a train of 1,600 action potentials delivered with an 80 Hz electric field stimulation (50 mA, 1-ms pulse width) in the imaging solution (144 mM NaCl, 2.5 mM KCl, 2.5 mM CaCl_2_, 2.5 mM MgCl_2_, 10 mM HEPES [pH 7.5], 10 μM CNQX, and 50 μM AP-5) [49] in the presence of 50 μM 40 kDa TMR-dextran (Invitrogen). Subsequently, neurons were perfused with the same buffer for 5 min to remove excess dextran dye. Experiments were performed at room temperature. Imaging was achieved through MetaMorph software and an ANDOR iXon 897 camera. Image processing was achieved using Image J and LSM Zen.

### Statistics

Paired and multiple data sets were compared by Student’s *t* test and one-way ANOVA statistical analyses, respectively. All data analyses were achieved using GraphPad Prism 7.0. The numerical data used in all figures are included in S1 Data.

## Supporting information

S1 Fig The expression level of *UAS*-*fwe* transgenes in NMJ boutons. (A-B) Confocal Z-projection images of NMJ boutons stained with α-HA (magenta) and a-HRP (green) were obtained from larvae expressing 50% Fwe (*nSyb* > *flag-fwe-HA* in *fwe*^*DB25*^*/fwe*^*DB56*^, A) or 50% FweE79Q (*nSyb > flag-fweE79Q-HA* in *fwe*^*DB25*^*/fwe*^*DB56*^, B). Type Ib and Is boutons are indicated. (C-F) Confocal Z-projection images of NMJ boutons stained with α- Fwe (magenta) and a-HRP (green) were derived from *FRT80B* control larvae (C, E), *fwe* mutant larvae *fwe*^*DB25*^*/fwe*^*DB56*^, D) and genomic *HA-fwe*-rescued larvae (*genomic HA-fwe construct/+*; *fwe*^*DB25*^*/fwe*^*DB56*^, F). Type Ib and Is boutons are indicated. In wild-type boutons, α-Fwe staining labels presynaptic compartments, whereas these staining signals are largely depleted upon loss of *fwe*, showing that this newly generated antibody is specific for Fwe. (L) α-Fwe staining signal intensity is normalized to a-HRP staining signal intensity. The values shown are normalized to the average value of *FRT80B* controls. The expression of HA-Fwe protein driven by *fwe cis* regulatory element was estimated as ~ 22% of the endogenous Fwe protein level. (G-K) Confocal Z-projection images of NMJ boutons stained with α-HA (magenta) and a-HRP (green) were derived from genomic *HA-fwe*-rescued larvae (*genomic HA-fwe construct/+*; *fwe*^*DB25*^*/fwe*^*DB56*^, G), 10% Fwe-rescued larvae (*elav* > *flag-fwe-HA* in *fwe*^*DB25*^*/fwe*^*DB56*^, H), 10% FweE79Q-rescued larvae (*elav* > *flag-fweE79Q-HA* in *fwe*^*DB25*^*/fwe*^*DB56*^, I), 4% Fwe-rescued larvae (*nSyb(w)* > *flag-fwe-HA* in *fwe*^*DB25*^*/fwe*^*DB56*^, J) or 4% FweE79Q-rescued larvae (*nSyb(w)* > *flag-fweE79Q-HA* in *fwe*^*DB25*^*/fwe*^*DB56*^, K). Type Ib and Is boutons are indicated. (M) α-HA staining signal intensity is normalized to a-HRP staining signal intensity. The values shown are normalized to the average value of genomic *HA-fwe*-rescued larvae. (N) % of the endogenous Fwe expression was calculated from the values shown in L and M. Type Ib boutons derived from A2/A3 muscles 6/7 were counted, and NMJs (control, n = 10; genomic HA-Fwe rescue, n = 8; 50% Fwe, n = 8; 50% FweE79Q, n = 8; 10% Fwe, n = 11; 10% FweE79Q, n = 8; 4% Fwe, n = 11; and 4% FweE79Q, n = 7) derived from at least five larvae for each genotype were analyzed. Student’s *t*-test was used for statistical analyses. *p*-Value: ns, not significant; ****, *p*<0.001. Error bars indicate the standard error of mean. Scale bar is 5 μm. The underlying data can be found in S1 Data.
(TIF)

S2 Fig CME is prevalently triggered by moderate K^+^ stimulation paradigms. (A) The experimental paradigm for the FM1-43 dye uptake assay upon mild stimulations. Larvae were dissected in 5 mM K^+^/0 mM Ca^2+^ HL-3 solution (resting condition) and then subjected to different stimulation paradigms in the presence of 4 mM fixable FM1-43 dye. After extensive washing, the samples were fixed with 4% paraformaldehyde for 10 min. FM1-43 dye-labeled NMJ boutons were imaged to indicate dye loading. (B-D) The confocal Z-projection images of NMJ boutons labeled with fixable FM1-43 dye were obtained from *FRT80B* larvae. FM1-43 dye uptake was evoked by the indicated conditions. (E) Data quantifications of the absolute unit (A.U.) of the FM1-43 dye fluorescence intensity. Under the resting condition, no FM1-43 dye uptake was observed. Either 1-min 90 mM K^+^/0.5 mM Ca^2+^ or 10-min 60 mM K^+^/1 mM Ca^2+^ stimulation causes similar efficiency of the dye uptake. Type Ib boutons derived from A2 muscles 6/7 were counted, and NMJs (1-min 90 mM K^+^/0.5 mM Ca^2+^, n = 8; and 10-min 60 mM K^+^/1 mM Ca^2+^, n = 6) derived from 5 larvae for each genotype were analyzed. Student’s *t*-test was used for statistical analysis. (F-H) TEM images of NMJ boutons were obtained from *FRT80B* control larvae. The samples were processed under the resting condition (10-min incubation in 5 mM K^+^/0 mM Ca^2+^ solution, F), 1-min 90 mM K^+^/0.5 mM Ca^2+^ stimulation (G) or 10-min 60 mM K^+^/1 mM Ca^2+^ stimulation (H). (I) Data quantifications of the number of bulk cisternae per bouton area. Type Ib boutons (10-min 5 mM K^+^/0 mM Ca^2+^, n = 17; 1-min 90 mM K^+^/0.5 mM Ca^2+^, n = 21; and 10-min 60 mM K^+^/1 mM Ca^2+^, n = 11) derived from at least three larvae for each genotype were analyzed. One-way ANOVA test was used for statistical analysis. *p*-Value: ns, not significant. Error bars indicate the standard error of mean. Scale bar: 5 μm in B-D; 500 nm in F-H. The underlying data can be found in S1 Data.
(TIF)

S3 Fig SV exocytosis is normal upon loss of *fwe* in high K^+^ stimulation. (A-E) The experimental paradigm for the FM1-43 dye loading/unloading assay upon 90 mM K^+^/0.5 mM Ca^2+^ stimulation (A). *FRT80B* control and *fwe* mutant larvae were dissected in 0 mM Ca^2+^ HL-3 solution and then subjected to 5-min 90 mM K^+^/0.5 mM Ca^2+^ stimulation, which releases SVs and induces endocytosis to load SVs with FM1-43 dye. Excess dye was removed by extensive washing of 0 mM Ca^2+^ HL-3 solution. The loaded dye in boutons was imaged to indicate “Loading” (B, D). Subsequently, the loaded dye in SVs was unloaded by 1-min 90 mM K^+^/0.5 mM Ca^2+^ stimulation. Released dye was washed out. The remaining dye in boutons was imaged to indicate “Unloading” (C, E). (F) The absolute unit of the dye fluorescence intensity in boutons was measured and normalized to the average value of controls. (G) The dye unloading efficiency was calculated from (F_load_-F_unload_)/F_load_. Both control and *fwe* mutant boutons release SVs in a similar rate. (H-N) The experimental paradigm for the FM1-43 dye loading/unloading assay upon 90 mM K^+^/2 mM Ca^2+^ stimulation (H). The experimental procedures and data quantifications are identical as the 90 mM K^+^/0.5 mM Ca^2+^ stimulation protocol. Under these conditions, the dye unloading efficiency in control and *fwe* mutant boutons is also comparable. Type Ib boutons derived from A2 muscles 6/7 were counted, and NMJs (90 mM K^+^/0.5 mM Ca^2+^: control, n = 18; and *fwe* mutant, n = 22. 90 mM K^+^/2 mM Ca^2+^: control, n = 24; and *fwe* mutant, n = 14) derived from at least four larvae for each genotype were analyzed. Student’s *t*-test was used for statistical analysis. *p*-Value: ns, not significant; ****, *p*<0.001. Error bars indicate the standard error of mean. All images were captured in the same scale. The underlying data can be found in S1 Data.
(TIF)

S4 Fig The expression of GCaMP6f in NMJ boutons. (A-D) Confocal Z-projection images of NMJ boutons were obtained from control larvae (*vglut-lexA/lexAop2-GCaMP6f*, *nSyb(w)-GAL4/+* in *fwe*^*DB25*^*/+*, A-A1), *fwe* mutant larvae (*vglut-lexA/lexAop2-GCaMP6f*, *nSyb(w)-GAL4/+* in *fwe*^*DB25*^*/fwe*^*DB56*^, B-B1), 4% Fwe-rescued larvae (*vglut-lexA/lexAop2-GCaMP6f*, *nSyb(w)-GAL4/ UAS-flag-fwe-RB-HA* in *fwe*^*DB25*^*/fwe*^*DB56*^, C-C1) and 4% FweE79Q-rescued larvae (*vglut-lexA/lexAop2-GCaMP6f*, *nSyb(w)-GAL4/UAS-flag-fweE79Q-RB-HA* in *fwe*^*DB25*^*/fwe*^*DB56*^, D-D1). The expression level of GCaMP6f in boutons was estimated by α-GFP staining signal intensity (red). Boutons were also stained for Disc large (Dlg, green), a postsynaptic marker. (E) α-GFP staining signal intensity is normalized to α-Dlg staining signal intensity. The values shown are normalized to the average value of controls. The level of GCaMP6f in *fwe* mutants is higher than that in other genotypes. Type Ib boutons derived from A2/3 muscles 6/7 were counted, and NMJs (control, n = 6; *fwe* mutant, n = 6; 4% Fwe, n = 11; and 4% FweE79Q, n = 10) derived from at least five larvae for each genotype were analyzed. (F-J) Confocal Z-projection images of NMJ boutons were obtained from control larvae(*nSyb*-*GAL4/UAS*-*GCaMP6f* in *fwe*^*DB25*^/*+*, F-F1), *fwe* mutant larvae (*nSyb*-*GAL4/UAS*-*GCaMP6f* in *fwe*^*DB25*^/*fwe*^*DB56*^, G-G1), 50% Fwe-rescued larvae (*nSyb*-*GAL4/UAS*-*GCaMP6f*/*UAS*-*flag-fwe-RB-HA* in *fwe*^*DB25*^*fwe*^*DB56*^, H-H1), 50% FweE79Q-rescued larvae(*nSyb*-*GAL4/UAS*-*GCaMP6f*/*UAS*-*flag-fweE79Q-RB-HA* in *fwe*^*DB25*^/*fwe*^*DB5*^, I-I1) and *dap160* mutant larvae (*nSyb*-*GAL4/UAS*-*GCaMP6f* in *dap160*^*Δ1*^/*dap160*^*Δ2*^, J-J1). Boutons were stained with α-GFP (red) and α-HRP (green). (K) α-GFP staining signal intensity is normalized to α-HRP staining signal intensity. The values shown are normalized to the average value of controls. The level of GCaMP6f in all genotypes is comparable. Type Ib boutons derived from A2/3 muscles 6/7 were counted, and NMJs (control, n = 10; *fwe* mutant, n = 10; 50% Fwe, n = 9; 50% FweE79Q, n = 7; and *dap160* mutant, n = 9) derived from at least five larvae for each genotype were analyzed. One-way ANOVA test was used for statistical analysis. *p*-Value: ns, not significant; *p*<0.05, **. Error bars indicate the standard error of mean. Scale bar is 5 μm. The underlying data can be found in S1 Data.
(TIF)

S5 Fig GCaMP6f responses upon electric stimulation. (A-E) Pseudocolored GCaMP6f images of NMJ boutons were obtained from control larvae(*nSyb*-*GAL4/UAS*-*GCaMP6f* in fwe^*DB25*^/*+*, A-A4), *fwe* mutant larvae (*nSyb*-*GAL4/UAS*-*GCaMP6f* in *fwe*^*DB25*^/*fwe*^*DB56*^, B-B4), 50% Fwe-rescued larvae(*nSyb*-*GAL4/UAS*-*GCaMP6f*/*UAS*-*flag-fwe-RB-HA* in *fwe*^*DB25*^/*fwe*^DB56^, C-C4), 50% Fwe-rescued larvae (*nSyb*-*GAL4/UAS*-*GCaMP6f*/*UAS*-*flag-fweE79Q-RB-HA* in *fwe*^*DB25*^/*fwe*^*DB56*^, D-D4) and *dap160* mutant larvae(*nSyb*-*GAL4/UAS*-*GCaMP6f* in *dap160*^*Δ1*^/*dap160*^*Δ2*^, E-E4). White arrows indicate type Ib boutons. Boutons were stimulated with trains of 10, 20 Hz and 40Hz-triggered action potentials, with 20-second rest between train stimuli. Representative GCaMP6f images were taken at the time points indicated by white arrows in Fig 3H. (F) The absolute unit (A.U.) of the resting GCaMP6f fluorescence intensity is shown. Loss of *fwe* impairs the resting Ca^2+^ levels, which is completely reversed when 50% Fwe is present. A subtle reduction in the resting Ca^2+^ levels was found in 50% FweE79Q-rescued boutons. *dap160* mutant boutons also display low basal Ca^2+^ concentrations. Type Ib boutons of A3 muscles 6/7 were counted, and NMJs (control, n = 17; *fwe* mutant, n = 17; 50% Fwe, n = 15; 50% FweE79Q, n = 18; and *dap160* mutant, n = 18) derived from at least six larvae for each genotype were analyzed. One-way ANOVA test were used for statistical analysis. *p*-Value: ns, not significant; *, *p*<0.05; ***, *p*<0.01; ****, *p*<0.001. Error bars indicate the standard error of mean. All images were captured in the same scale.
(TIF)

S6 Fig The expression and distribution of Cacophony is normal in *fwe* mutants. (A-B) The single-section confocal images of NMJ boutons stained for Bruchpilot, an active zone scaffolding protein (with nc82 antibody, magenta), Cac-EGFP (with α-GFP, green) and neuronal membrane (with a-HRP, blue) were derived from control larvae (*nSyb* > *cac-EGFP* in *fwe*^*DB25*^/+, A-A3) and *fwe* mutant larvae (*nSyb* > *cac-EGFP* in *fwe*^*DB25*^/*fwe*^*DB56*^, B-B3). Individual type Ib boutons are outlined with white dashed lines based on a-HRP staining signal (blue). White arrows indicate Cac-GFP signals that are associated with the active zones, and white arrow heads indicates Cac-GFP signals that are not associated with active zones. Scale bar: 1 μm.
(TIF)

S7 Fig 50% FweE79Q can induce ADBE in high K^+^ stimulation. (A-B) TEM images of NMJ boutons were obtained from 50% FweE79Q-rescued larvae (*nSyb* > *flag-fweE79Q-HA* in *fwe*^*DB25*^/*fwe*^*DB56*^) that were fixed under the resting condition (10-min incubation in 5 mM K^+^/0 mM Ca^2+^ solution, A) or after 10-min 90 mM K^+^/2 mM Ca^2+^ stimulation (B). Bulk cisternae larger than 80 nm are indicated by red arrows. (C) Data quantifications of the number of bulk cisternae per bouton area. Type Ib boutons (at rest, n = 17; and 10-min 90 mM K^+^/2 mM Ca^2+^, n = 20) derived from three larvae for each genotype were analyzed. Student’s *t*-test was used for statistical analysis. *p*-Value: ****, *p*<0.001. Error bars indicate the standard error of mean. Scale bar: 500 nm. The underlying data can be found in S1 Data.
(TIF)

S8 Fig The gene structure of *mFwe*. Schematic representation of *Cacfd1* (*mFwe*) gene locus composed of the *UTR* regions (white boxes), the coding exons (red boxes) and the introns (black lines). 6 alternative mRNA splicing isoforms (corresponding transcript numbers are indicated in right) are predicted to generate at least 5 protein isoforms indicated in bottom box. Predicted protein topology is indicated. Putative Ca^2+^-binding residue (glutamic acid, E) in the transmembrane domain is highlighted. mFwe2 (red) is the most similar to *Drosophila* Fwe.
(TIF)

S9 Fig The expression and subcellular localization of rodent Fwe2. (A) In the immunoblot, mFwe2 is present in normal mouse neuroblastoma n2a cells but absent in *mFwe* knockout n2a cells. Tubulin was used as the loading control. (B) Confocal Z-projection images of DIV14 cultured rat hippocampal neurons stained with α-m/ratFwe2 (red), a-GM130 (magenta), DAPI (blue) and α-GFP (green) were captured from neurons transfected with *pSpCas9(BB)-2A-GFP* plasmid. RatFwe2 is highly colocalized with GM130, a *cis*-Golgi marker, in the cell bodies outlined by α-GFP staining. (C-E) Confocal Z-projection images of DIV14 cultured rat hippocampal neurons were captured from neurons transfected with *pSpCas9(BB)-2A-GFP* (C-C1), *pSpCas9(BB)-m/ratFwe-gRNA-2A-GFP* (D-D1) or *pSpCas9(BB)-m/ratFwe-gRNA-2A-GFP-2A-mFwe2-HA* (E-E1) plasmid. Neurons shown in C-D were stained with α-m/ratFwe2 (red), α-GFP (green) and DAPI (blue). The neuron shown in E was stained with α-HA (magenta), α-GFP (green) and DAPI (blue). RatFwe in the cell body is dramatically decreased in the presence of Cas9 and m/ratFwe-gRNA. Scale bar: 10 μm.
(TIF)

S10 Fig Loss of *ratFwe2* does not affect SV re-acidification. (A) The time-course traces of Synaptophysin-phluorin (SypHy) fluorescence in the presynaptic terminals of DIV13-15 cultured rat hippocampal neurons. Neurons expressing *pSpCas9(BB)-2A-tagRFP*/*pCMV-Syphy* (control, black line) or *pSpCas9(BB)-m/ratFwe-gRNA-2A-tagRFP/ pCMV-Syphy* (m/ratFwe-gRNA, red line) plasmids were stimulated with a train of 200 APs evoked at 20 Hz in the imaging solution (pH 7.4). SV exocytosis causes the increases in SypHy fluorescence. In turn, SV endocytosis and re-acidification leads to fluorescence decays. Neurons were then subjected to 22-second perfusion with an acidic buffer (pH 5.5), by which the fluorescence of all surface-associated SypHy proteins is quenched, but SypHy associated with newly formed SVs is initially resistant and gradually quenched by H^+^ pump-driven acidification (dashed box). The recording bath was in turn perfused with pH 7.4 imaging solution, by which the originally quenched surface-associated SypHy becomes fluorescent. (B) The time-course traces for SypHy fluorescence changes during SV re-acidification (dashed box in A) are enlarged. Both controls and *ratfwe* knockouts display similar SV re-acidification rate. Presynaptic terminals (control, n = 19; and m/ratFwe-gRNA, n = 22) co-labeled with TagRFP and SypHy derived from at least five coverslip cultures were analyzed. Student’s *t*-test was used for paired comparisons. *p*-Value: ns, not significant. Error bars are the standard error of mean. The underlying data can be found in S1 Data.
(TIF)

S1 Data Excel datasheet containing the numerical value and statistical analysis for Figure panels 1H, 1Q, 1R, 2G, 2H, 2I, 2J, 2M, 2N, 3E, 3F, 3G, 3H, 3I, 4K, 4L, 4M, 4P, 5E, 5F, 5I, 6K, 6N, 7A, 7B, 7C, 7D, 7H, S1L, S1M, S1N, S2E, S2I, S3F, S3G, S3M, S3N, S4E, S4K, S5F, S7C, S10A, S10B. (XLSX)

## Abbreviations

ADBE: activity-dependent bulk endocytosis
A.U.: absolute unit
CME: Clathrin-mediated endocytosis
CRAC: calcium release-activated channel
CRISPR/Cas9: clustered regularly interspaced short palindromic repeats/ CRISPR-associated protein 9
DIV: days in vitro
EJP: excitatory junction potential
Fwe: Flower
GFP: green fluorescent protein
gRNA: guide RNA
HL: hemolymph-like
HRP: horse radish peroxidase
LP1: lysate pellet 1
LP2: lysate pellet 2
LS2: lysate supernatant 2
miRNAi: microRNAi
n2a: Neuro 2a
NMJ: neuromuscular junction
RT-PCR: reverse-transcription PCR
SNARE: soluble NSF attachment protein receptor
SV: synaptic vesicle
Syp: Synaptophysin
SypHy: Synaptophysin-pHluorin
TEM: transmission electron microscopy
TMR: tetramethylrhodamine
TRPV: transient receptor potential cation channel subfamily V member
VAMP4: vesicle-associated membrane protein 4
VGCC: voltage-gated Ca^2+^ channel
v-SNARE: vesicle-associated SNARE
