# Supplementary figures and images for: A Ca2+ channel differentially regulates Clathrin-mediated and activity-dependent bulk endocytosis
Source: PLoS Biol. 2017 Apr 17;15(4):e2000931. doi: 10.1371/journal.pbio.2000931 (PMC5393565; doi:10.1371/journal.pbio.2000931)

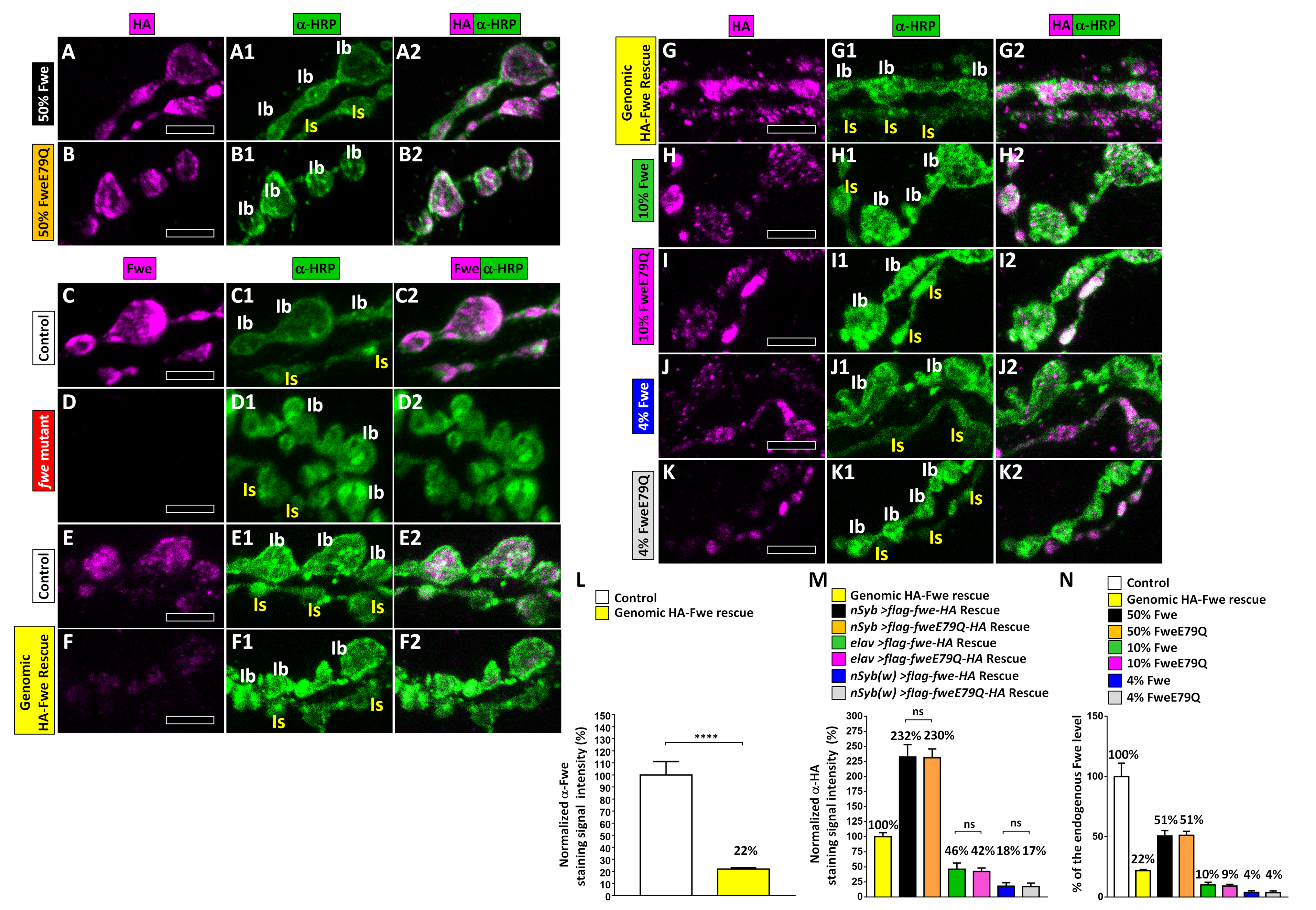

Supplement: S1 Fig — (A-B) Confocal Z-projection images of NMJ boutons stained with α-HA (magenta) and a-HRP (green) were obtained from larvae expressing 50% Fwe (nSyb > flag-fwe-HA in fweDB25/fweDB56, A) or 50% FweE79Q (nSyb > flag-fweE79Q-HA in fweDB25/fweDB56, B). Type Ib and Is boutons are indicated. (C-F) Confocal Z-projection images of NMJ boutons stained with α- Fwe (magenta) and a-HRP (green) were derived from FRT80B control larvae (C, E), fwe mutant larvae fweDB25/fweDB56, D) and genomic HA-fwe-rescued larvae (genomic HA-fwe construct/+; fweDB25/fweDB56, F). Type Ib and Is boutons are indicated. In wild-type boutons, α-Fwe staining labels presynaptic compartments, whereas these staining signals are largely depleted upon loss of fwe, showing that this newly generated antibody is specific for Fwe. (L) α-Fwe staining signal intensity is normalized to a-HRP staining signal intensity. The values shown are normalized to the average value of FRT80B controls. The expression of HA-Fwe protein driven by fwe cis regulatory element was estimated as ~ 22% of the endogenous Fwe protein level. (G-K) Confocal Z-projection images of NMJ boutons stained with α-HA (magenta) and a-HRP (green) were derived from genomic HA-fwe-rescued larvae (genomic HA-fwe construct/+; fweDB25/fweDB56, G), 10% Fwe-rescued larvae (elav > flag-fwe-HA in fweDB25/fweDB56, H), 10% FweE79Q-rescued larvae (elav > flag-fweE79Q-HA in fweDB25/fweDB56, I), 4% Fwe-rescued larvae (nSyb(w) > flag-fwe-HA in fweDB25/fweDB56, J) or 4% FweE79Q-rescued larvae (nSyb(w) > flag-fweE79Q-HA in fweDB25/fweDB56, K). Type Ib and Is boutons are indicated. (M) α-HA staining signal intensity is normalized to a-HRP staining signal intensity. The values shown are normalized to the average value of genomic HA-fwe-rescued larvae. (N) % of the endogenous Fwe expression was calculated from the values shown in L and M. Type Ib boutons derived from A2/A3 muscles 6/7 were counted, and NMJs (control, n = 10; genomic HA-Fwe rescue, n = 8; 50% Fwe, n = 8; [file pbio.2000931.s001.tif]

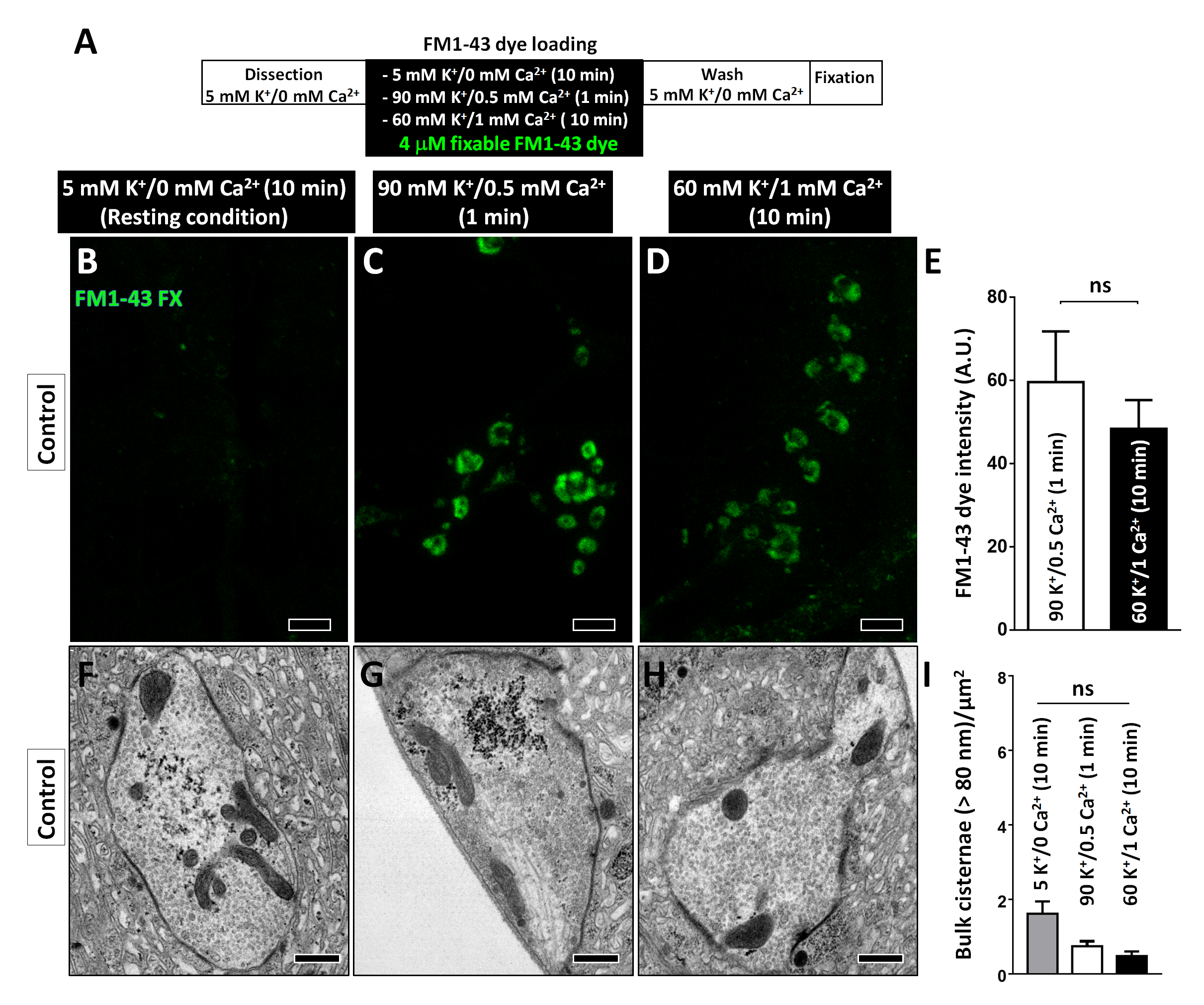

Supplement: S2 Fig — (A) The experimental paradigm for the FM1-43 dye uptake assay upon mild stimulations. Larvae were dissected in 5 mM K+/0 mM Ca2+ HL-3 solution (resting condition) and then subjected to different stimulation paradigms in the presence of 4 mM fixable FM1-43 dye. After extensive washing, the samples were fixed with 4% paraformaldehyde for 10 min. FM1-43 dye-labeled NMJ boutons were imaged to indicate dye loading. (B-D) The confocal Z-projection images of NMJ boutons labeled with fixable FM1-43 dye were obtained from FRT80B larvae. FM1-43 dye uptake was evoked by the indicated conditions. (E) Data quantifications of the absolute unit (A.U.) of the FM1-43 dye fluorescence intensity. Under the resting condition, no FM1-43 dye uptake was observed. Either 1-min 90 mM K+/0.5 mM Ca2+ or 10-min 60 mM K+/1 mM Ca2+ stimulation causes similar efficiency of the dye uptake. Type Ib boutons derived from A2 muscles 6/7 were counted, and NMJs (1-min 90 mM K+/0.5 mM Ca2+, n = 8; and 10-min 60 mM K+/1 mM Ca2+, n = 6) derived from 5 larvae for each genotype were analyzed. Student’s t-test was used for statistical analysis. (F-H) TEM images of NMJ boutons were obtained from FRT80B control larvae. The samples were processed under the resting condition (10-min incubation in 5 mM K+/0 mM Ca2+ solution, F), 1-min 90 mM K+/0.5 mM Ca2+ stimulation (G) or 10-min 60 mM K+/1 mM Ca2+ stimulation (H). (I) Data quantifications of the number of bulk cisternae per bouton area. Type Ib boutons (10-min 5 mM K+/0 mM Ca2+, n = 17; 1-min 90 mM K+/0.5 mM Ca2+, n = 21; and 10-min 60 mM K+/1 mM Ca2+, n = 11) derived from at least three larvae for each genotype were analyzed. One-way ANOVA test was used for statistical analysis. p-Value: ns, not significant. Error bars indicate the standard error of mean. Scale bar: 5 μm in B-D; 500 nm in F-H. The underlying data can be found in S1 Data. (TIF) [file pbio.2000931.s002.tif]

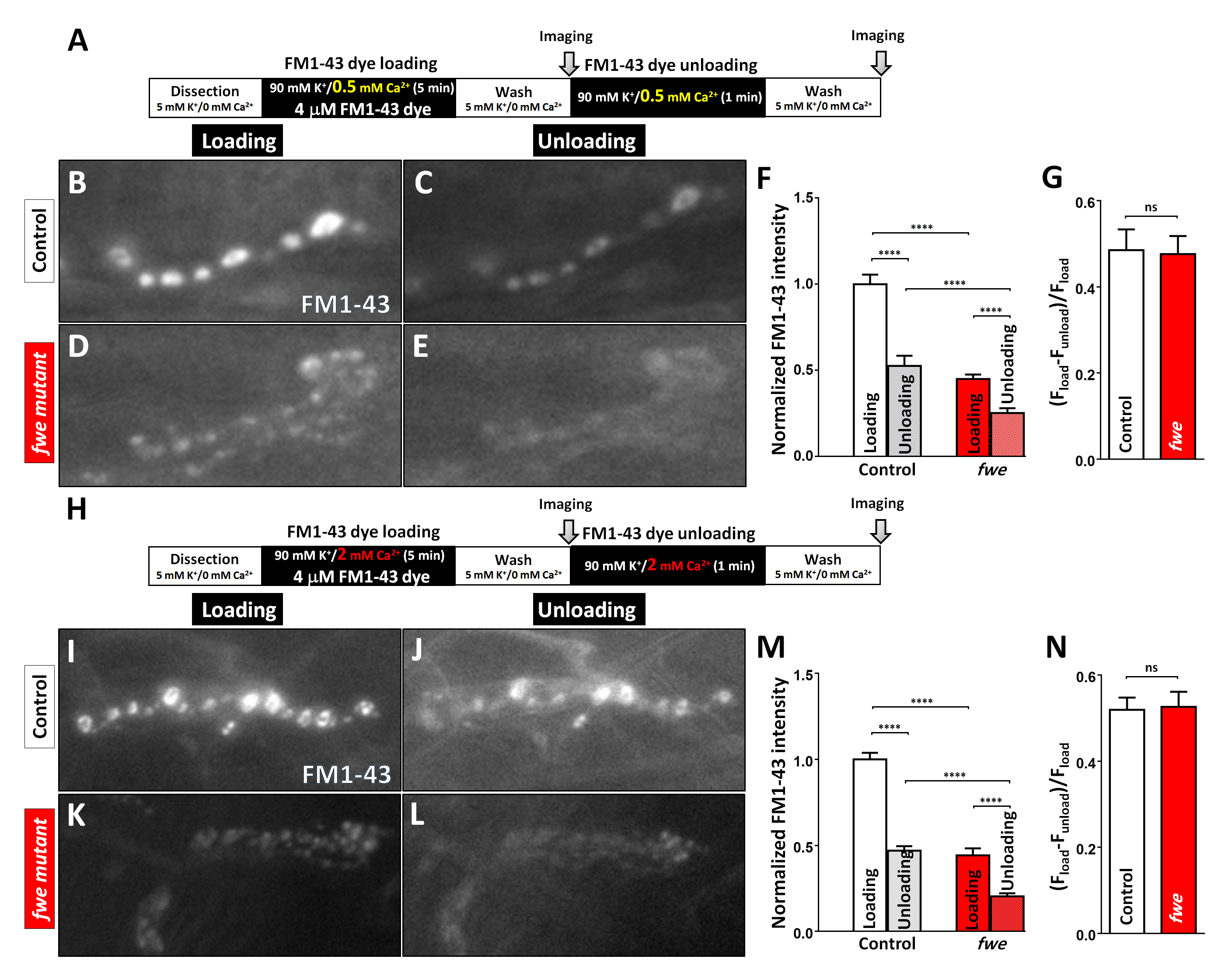

Supplement: S3 Fig — (A-E) The experimental paradigm for the FM1-43 dye loading/unloading assay upon 90 mM K+/0.5 mM Ca2+ stimulation (A). FRT80B control and fwe mutant larvae were dissected in 0 mM Ca2+ HL-3 solution and then subjected to 5-min 90 mM K+/0.5 mM Ca2+ stimulation, which releases SVs and induces endocytosis to load SVs with FM1-43 dye. Excess dye was removed by extensive washing of 0 mM Ca2+ HL-3 solution. The loaded dye in boutons was imaged to indicate “Loading” (B, D). Subsequently, the loaded dye in SVs was unloaded by 1-min 90 mM K+/0.5 mM Ca2+ stimulation. Released dye was washed out. The remaining dye in boutons was imaged to indicate “Unloading” (C, E). (F) The absolute unit of the dye fluorescence intensity in boutons was measured and normalized to the average value of controls. (G) The dye unloading efficiency was calculated from (Fload-Funload)/Fload. Both control and fwe mutant boutons release SVs in a similar rate. (H-N) The experimental paradigm for the FM1-43 dye loading/unloading assay upon 90 mM K+/2 mM Ca2+ stimulation (H). The experimental procedures and data quantifications are identical as the 90 mM K+/0.5 mM Ca2+ stimulation protocol. Under these conditions, the dye unloading efficiency in control and fwe mutant boutons is also comparable. Type Ib boutons derived from A2 muscles 6/7 were counted, and NMJs (90 mM K+/0.5 mM Ca2+: control, n = 18; and fwe mutant, n = 22. 90 mM K+/2 mM Ca2+: control, n = 24; and fwe mutant, n = 14) derived from at least four larvae for each genotype were analyzed. Student’s t-test was used for statistical analysis. p-Value: ns, not significant; ****, p<0.001. Error bars indicate the standard error of mean. All images were captured in the same scale. The underlying data can be found in S1 Data. (TIF) [file pbio.2000931.s003.tif]

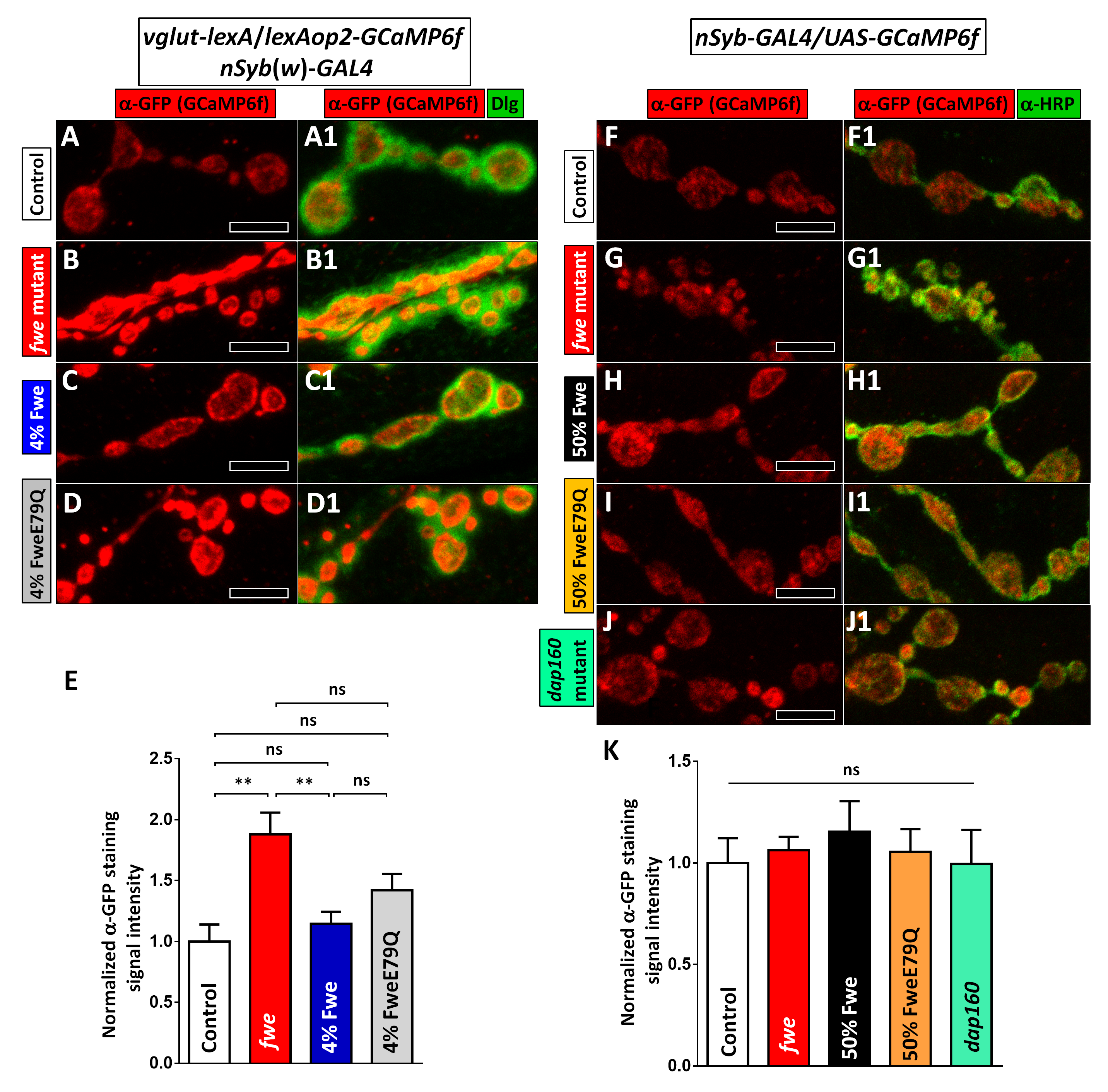

Supplement: S4 Fig — (A-D) Confocal Z-projection images of NMJ boutons were obtained from control larvae (vglut-lexA/lexAop2-GCaMP6f, nSyb(w)-GAL4/+ in fweDB25/+, A-A1), fwe mutant larvae (vglut-lexA/lexAop2-GCaMP6f, nSyb(w)-GAL4/+ in fweDB25/fweDB56, B-B1), 4% Fwe-rescued larvae (vglut-lexA/lexAop2-GCaMP6f, nSyb(w)-GAL4/ UAS-flag-fwe-RB-HA in fweDB25/fweDB56, C-C1) and 4% FweE79Q-rescued larvae (vglut-lexA/lexAop2-GCaMP6f, nSyb(w)-GAL4/UAS-flag-fweE79Q-RB-HA in fweDB25/fweDB56, D-D1). The expression level of GCaMP6f in boutons was estimated by α-GFP staining signal intensity (red). Boutons were also stained for Disc large (Dlg, green), a postsynaptic marker. (E) α-GFP staining signal intensity is normalized to α-Dlg staining signal intensity. The values shown are normalized to the average value of controls. The level of GCaMP6f in fwe mutants is higher than that in other genotypes. Type Ib boutons derived from A2/3 muscles 6/7 were counted, and NMJs (control, n = 6; fwe mutant, n = 6; 4% Fwe, n = 11; and 4% FweE79Q, n = 10) derived from at least five larvae for each genotype were analyzed. (F-J) Confocal Z-projection images of NMJ boutons were obtained from control larvae(nSyb-GAL4/UAS-GCaMP6f in fweDB25/+, F-F1), fwe mutant larvae (nSyb-GAL4/UAS-GCaMP6f in fweDB25/fweDB56, G-G1), 50% Fwe-rescued larvae (nSyb-GAL4/UAS-GCaMP6f/UAS-flag-fwe-RB-HA in fweDB25fweDB56, H-H1), 50% FweE79Q-rescued larvae(nSyb-GAL4/UAS-GCaMP6f/UAS-flag-fweE79Q-RB-HA in fweDB25/fweDB5, I-I1) and dap160 mutant larvae (nSyb-GAL4/UAS-GCaMP6f in dap160Δ1/dap160Δ2, J-J1). Boutons were stained with α-GFP (red) and α-HRP (green). (K) α-GFP staining signal intensity is normalized to α-HRP staining signal intensity. The values shown are normalized to the average value of controls. The level of GCaMP6f in all genotypes is comparable. Type Ib boutons derived from A2/3 muscles 6/7 were counted, and NMJs (control, n = 10; fwe mutant, n = 10; 50% Fwe, n = 9; 50% FweE79Q, n = 7; and dap160 mutant, n = 9) derived from at least [file pbio.2000931.s004.tif]

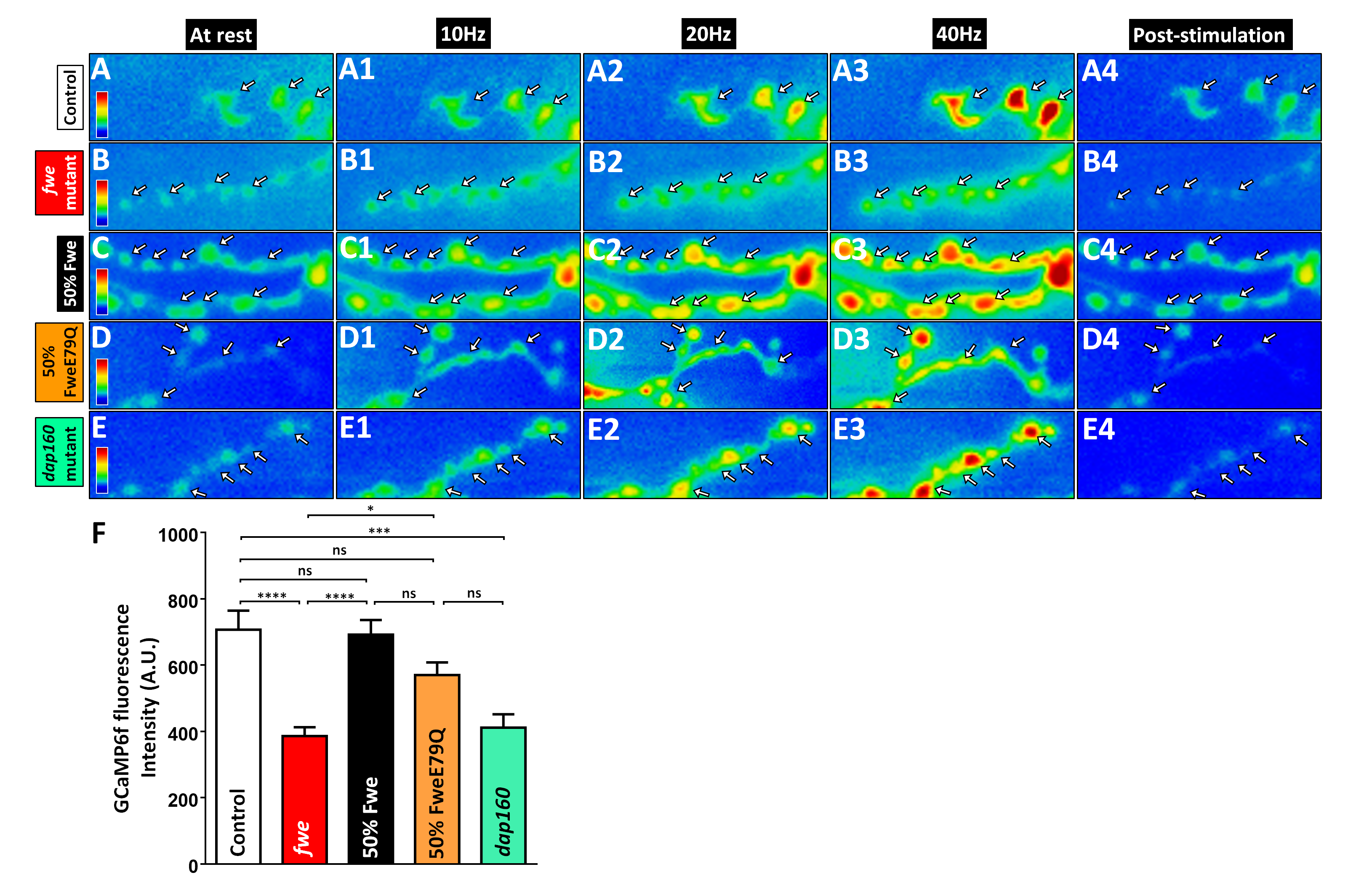

Supplement: S5 Fig — (A-E) Pseudocolored GCaMP6f images of NMJ boutons were obtained from control larvae(nSyb-GAL4/UAS-GCaMP6f in fweDB25/+, A-A4), fwe mutant larvae (nSyb-GAL4/UAS-GCaMP6f in fweDB25/fweDB56, B-B4), 50% Fwe-rescued larvae(nSyb-GAL4/UAS-GCaMP6f/UAS-flag-fwe-RB-HA in fweDB25/fweDB56, C-C4), 50% Fwe-rescued larvae (nSyb-GAL4/UAS-GCaMP6f/UAS-flag-fweE79Q-RB-HA in fweDB25/fweDB56, D-D4) and dap160 mutant larvae(nSyb-GAL4/UAS-GCaMP6f in dap160Δ1/dap160Δ2, E-E4). White arrows indicate type Ib boutons. Boutons were stimulated with trains of 10, 20 Hz and 40Hz-triggered action potentials, with 20-second rest between train stimuli. Representative GCaMP6f images were taken at the time points indicated by white arrows in Fig 3H. (F) The absolute unit (A.U.) of the resting GCaMP6f fluorescence intensity is shown. Loss of fwe impairs the resting Ca2+ levels, which is completely reversed when 50% Fwe is present. A subtle reduction in the resting Ca2+ levels was found in 50% FweE79Q-rescued boutons. dap160 mutant boutons also display low basal Ca2+ concentrations. Type Ib boutons of A3 muscles 6/7 were counted, and NMJs (control, n = 17; fwe mutant, n = 17; 50% Fwe, n = 15; 50% FweE79Q, n = 18; and dap160 mutant, n = 18) derived from at least six larvae for each genotype were analyzed. One-way ANOVA test were used for statistical analysis. p-Value: ns, not significant; *, p<0.05; ***, p<0.01; ****, p<0.001. Error bars indicate the standard error of mean. All images were captured in the same scale. (TIF) [file pbio.2000931.s005.tif]

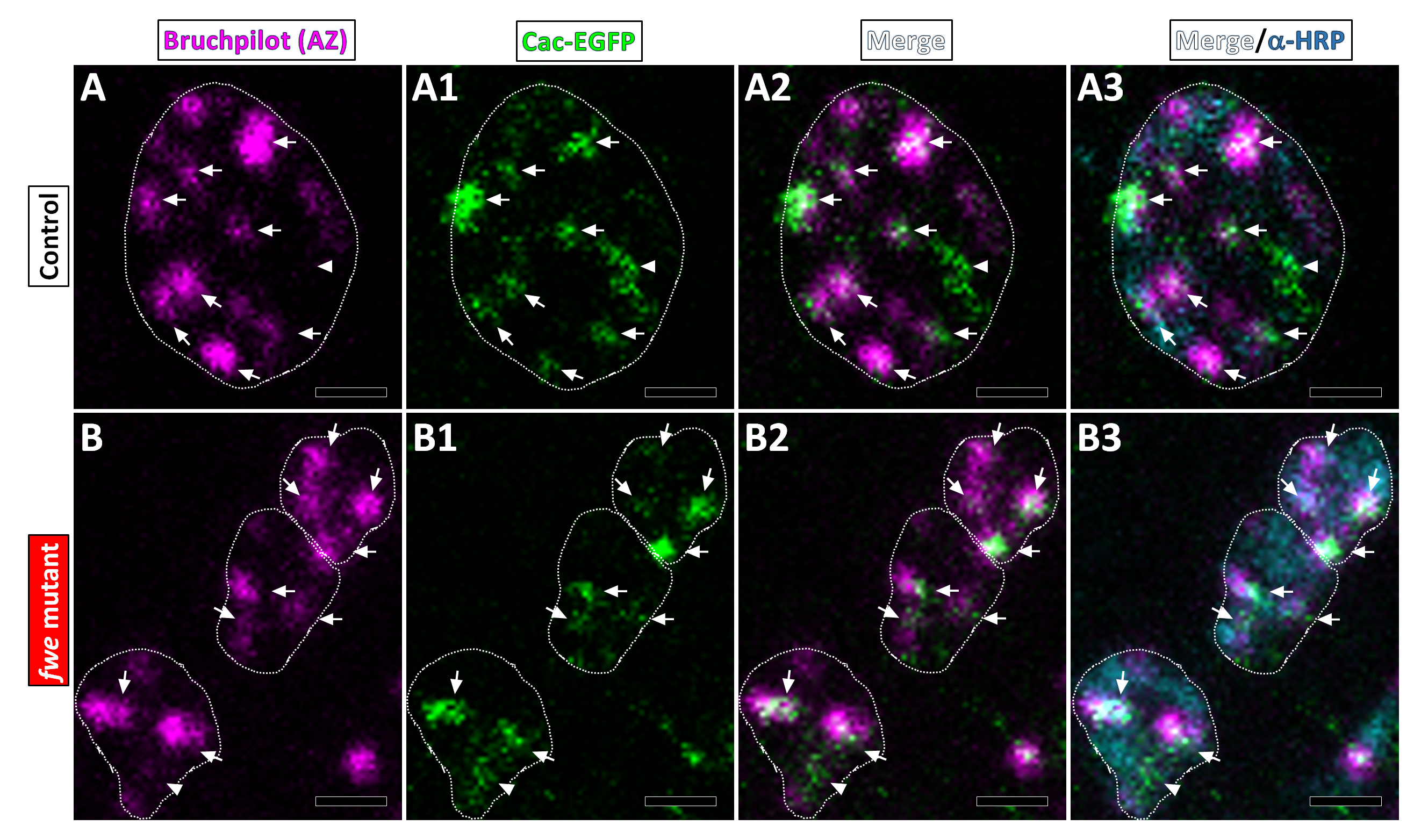

Supplement: S6 Fig — (A-B) The single-section confocal images of NMJ boutons stained for Bruchpilot, an active zone scaffolding protein (with nc82 antibody, magenta), Cac-EGFP (with α-GFP, green) and neuronal membrane (with a-HRP, blue) were derived from control larvae (nSyb > cac-EGFP in fweDB25/+, A-A3) and fwe mutant larvae (nSyb > cac-EGFP in fweDB25/fweDB56, B-B3). Individual type Ib boutons are outlined with white dashed lines based on a-HRP staining signal (blue). White arrows indicate Cac-GFP signals that are associated with the active zones, and white arrow heads indicates Cac-GFP signals that are not associated with active zones. Scale bar: 1 μm. (TIF) [file pbio.2000931.s006.tif]

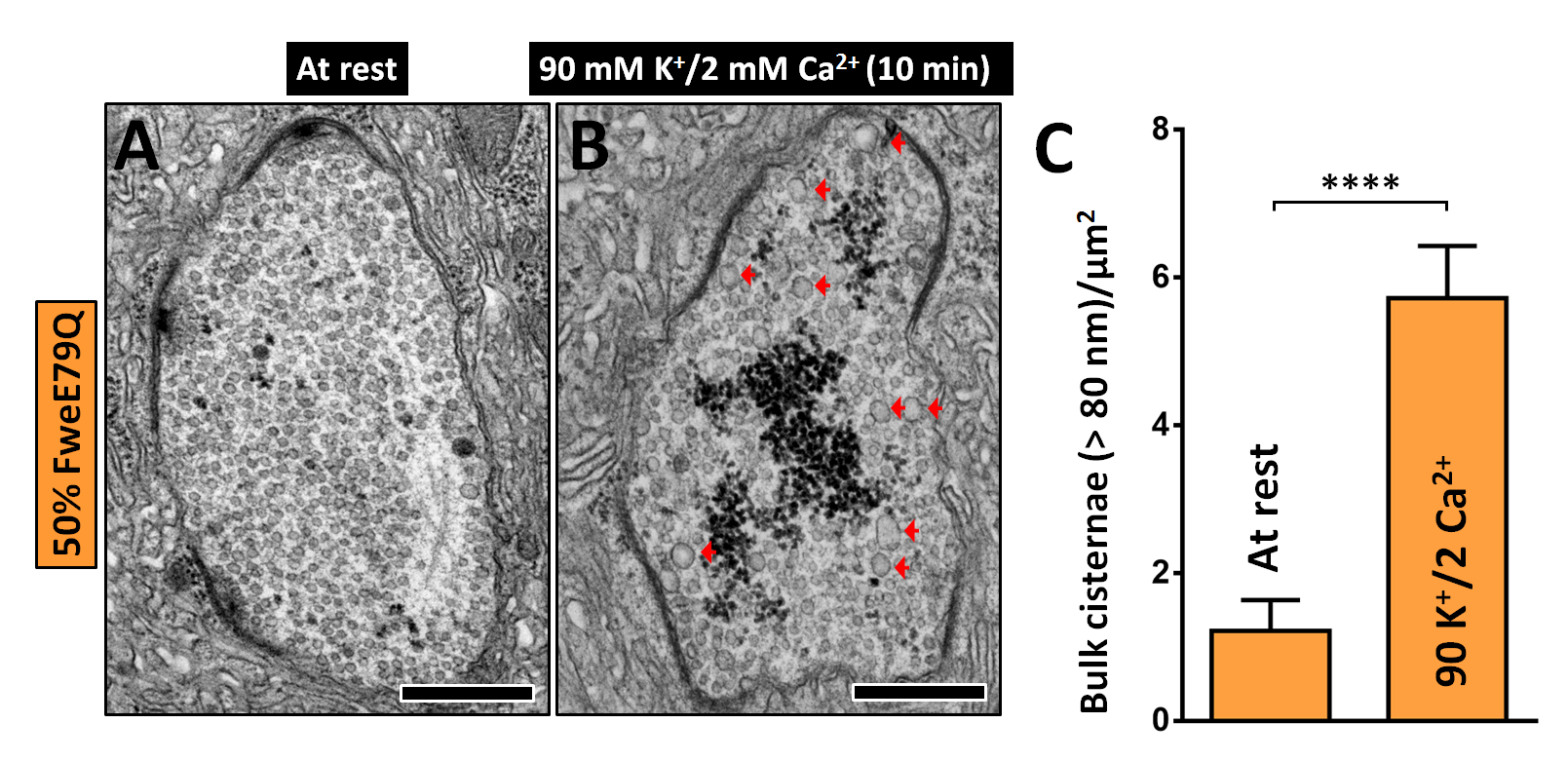

Supplement: S7 Fig — (A-B) TEM images of NMJ boutons were obtained from 50% FweE79Q-rescued larvae (nSyb > flag-fweE79Q-HA in fweDB25/fweDB56) that were fixed under the resting condition (10-min incubation in 5 mM K+/0 mM Ca2+ solution, A) or after 10-min 90 mM K+/2 mM Ca2+ stimulation (B). Bulk cisternae larger than 80 nm are indicated by red arrows. (C) Data quantifications of the number of bulk cisternae per bouton area. Type Ib boutons (at rest, n = 17; and 10-min 90 mM K+/2 mM Ca2+, n = 20) derived from three larvae for each genotype were analyzed. Student’s t-test was used for statistical analysis. p-Value: ****, p<0.001. Error bars indicate the standard error of mean. Scale bar: 500 nm. The underlying data can be found in S1 Data. (TIF) [file pbio.2000931.s007.tif]

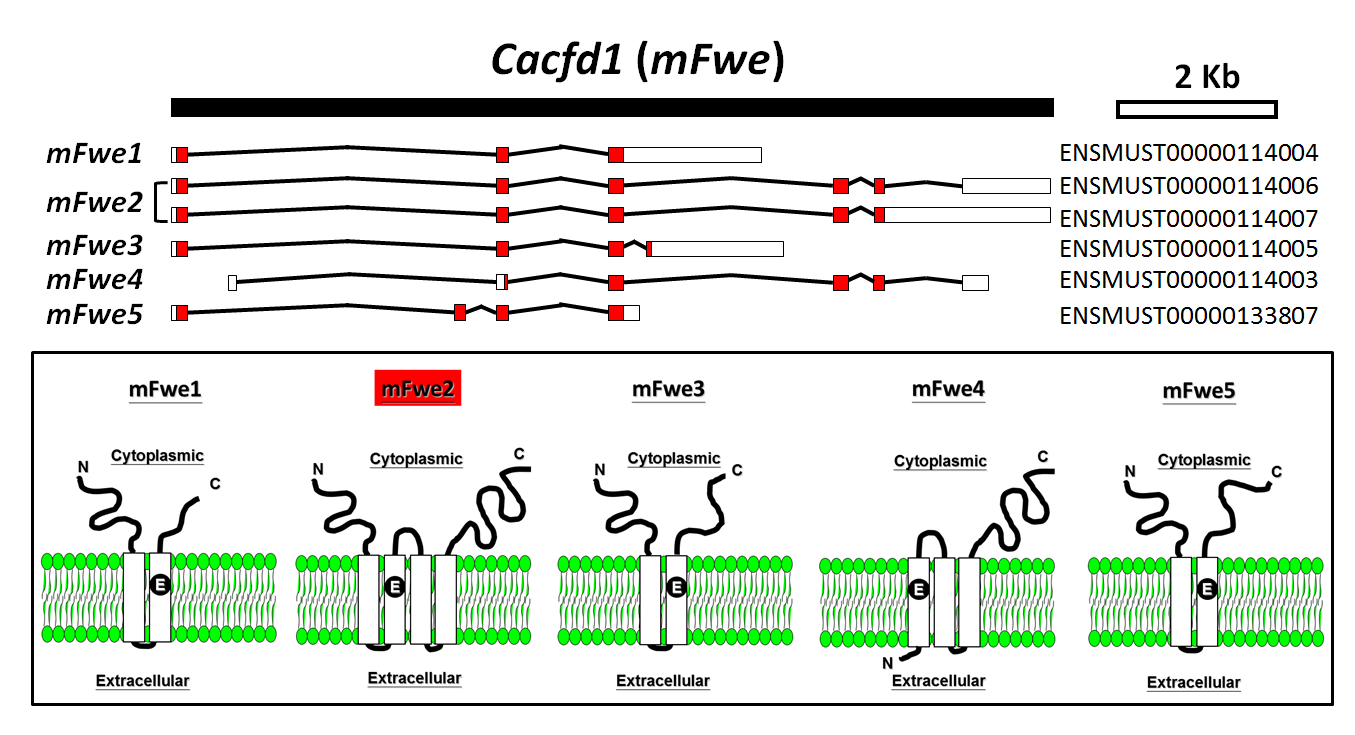

Supplement: S8 Fig — Schematic representation of Cacfd1 (mFwe) gene locus composed of the UTR regions (white boxes), the coding exons (red boxes) and the introns (black lines). 6 alternative mRNA splicing isoforms (corresponding transcript numbers are indicated in right) are predicted to generate at least 5 protein isoforms indicated in bottom box. Predicted protein topology is indicated. Putative Ca2+-binding residue (glutamic acid, E) in the transmembrane domain is highlighted. mFwe2 (red) is the most similar to Drosophila Fwe. (TIF) [file pbio.2000931.s008.tif]

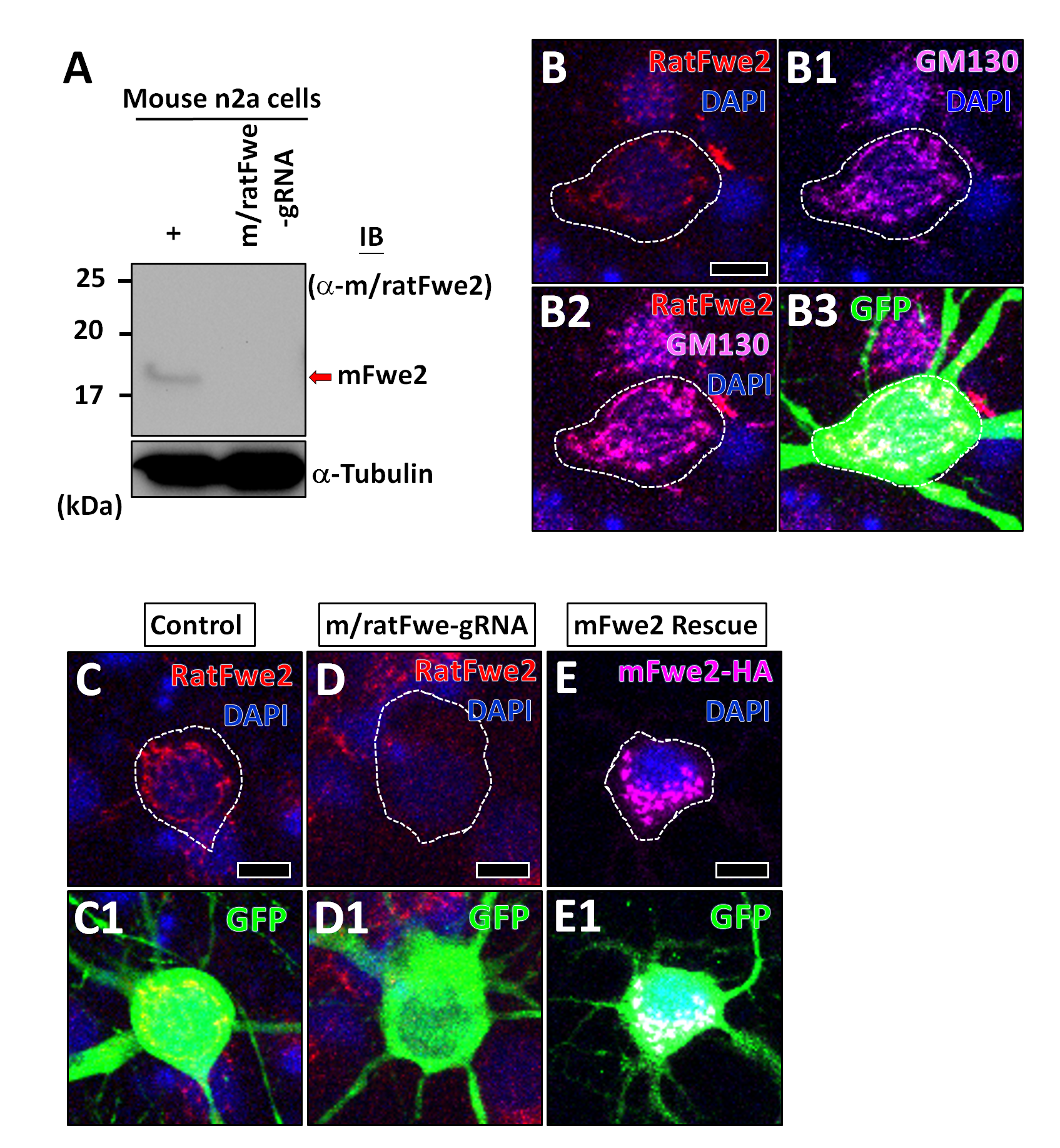

Supplement: S9 Fig — (A) In the immunoblot, mFwe2 is present in normal mouse neuroblastoma n2a cells but absent in mFwe knockout n2a cells. Tubulin was used as the loading control. (B) Confocal Z-projection images of DIV14 cultured rat hippocampal neurons stained with α-m/ratFwe2 (red), a-GM130 (magenta), DAPI (blue) and α-GFP (green) were captured from neurons transfected with pSpCas9(BB)-2A-GFP plasmid. RatFwe2 is highly colocalized with GM130, a cis-Golgi marker, in the cell bodies outlined by α-GFP staining. (C-E) Confocal Z-projection images of DIV14 cultured rat hippocampal neurons were captured from neurons transfected with pSpCas9(BB)-2A-GFP (C-C1), pSpCas9(BB)-m/ratFwe-gRNA-2A-GFP (D-D1) or pSpCas9(BB)-m/ratFwe-gRNA-2A-GFP-2A-mFwe2-HA (E-E1) plasmid. Neurons shown in C-D were stained with α-m/ratFwe2 (red), α-GFP (green) and DAPI (blue). The neuron shown in E was stained with α-HA (magenta), α-GFP (green) and DAPI (blue). RatFwe in the cell body is dramatically decreased in the presence of Cas9 and m/ratFwe-gRNA. Scale bar: 10 μm. (TIF) [file pbio.2000931.s009.tif]

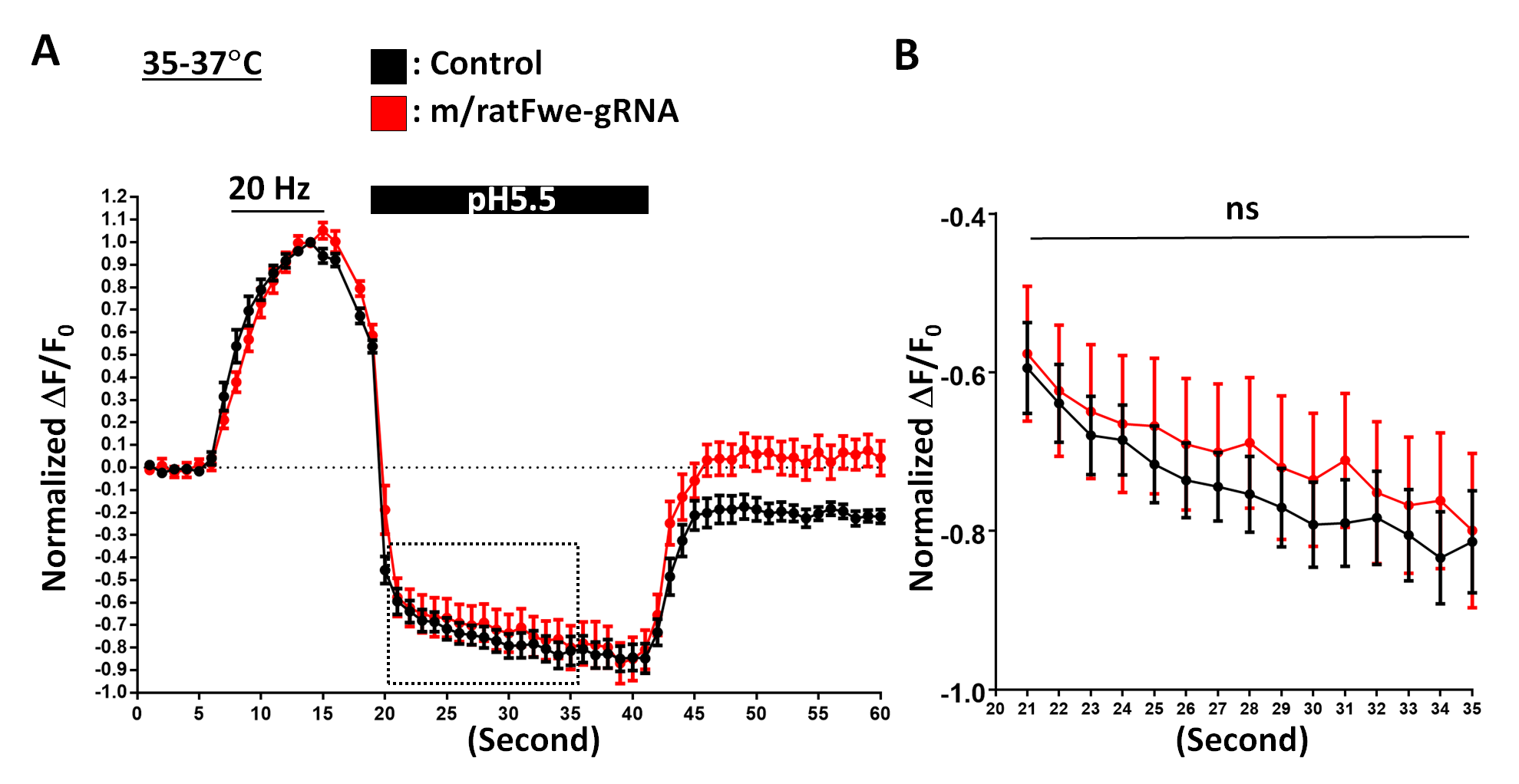

Supplement: S10 Fig — (A) The time-course traces of Synaptophysin-phluorin (SypHy) fluorescence in the presynaptic terminals of DIV13-15 cultured rat hippocampal neurons. Neurons expressing pSpCas9(BB)-2A-tagRFP/pCMV-Syphy (control, black line) or pSpCas9(BB)-m/ratFwe-gRNA-2A-tagRFP/ pCMV-Syphy (m/ratFwe-gRNA, red line) plasmids were stimulated with a train of 200 APs evoked at 20 Hz in the imaging solution (pH 7.4). SV exocytosis causes the increases in SypHy fluorescence. In turn, SV endocytosis and re-acidification leads to fluorescence decays. Neurons were then subjected to 22-second perfusion with an acidic buffer (pH 5.5), by which the fluorescence of all surface-associated SypHy proteins is quenched, but SypHy associated with newly formed SVs is initially resistant and gradually quenched by H+ pump-driven acidification (dashed box). The recording bath was in turn perfused with pH 7.4 imaging solution, by which the originally quenched surface-associated SypHy becomes fluorescent. (B) The time-course traces for SypHy fluorescence changes during SV re-acidification (dashed box in A) are enlarged. Both controls and ratfwe knockouts display similar SV re-acidification rate. Presynaptic terminals (control, n = 19; and m/ratFwe-gRNA, n = 22) co-labeled with TagRFP and SypHy derived from at least five coverslip cultures were analyzed. Student’s t-test was used for paired comparisons. p-Value: ns, not significant. Error bars are the standard error of mean. The underlying data can be found in S1 Data. (TIF) [file pbio.2000931.s010.tif]
